# Mitigating the risk of cytokine release syndrome in a Phase I trial of CD20/CD3 bispecific antibody mosunetuzumab in NHL: impact of translational system modeling

**DOI:** 10.1038/s41540-020-00145-7

**Published:** 2020-08-28

**Authors:** Iraj Hosseini, Kapil Gadkar, Eric Stefanich, Chi-Chung Li, Liping L. Sun, Yu-Waye Chu, Saroja Ramanujan

**Affiliations:** Genentech Inc., 1 DNA Way, South San Francisco, CA 94080 USA

**Keywords:** Cancer, Dynamical systems, Pharmacodynamics, Pharmacokinetics, Immunology

## Abstract

Mosunetuzumab, a T-cell dependent bispecific antibody that binds CD3 and CD20 to drive T-cell mediated B-cell killing, is currently being tested in non-Hodgkin lymphoma. However, potent immune stimulation with T-cell directed therapies poses the risk of cytokine release syndrome, potentially limiting dose and utility. To understand mechanisms behind safety and efficacy and explore safety mitigation strategies, we developed a novel mechanistic model of immune and antitumor responses to the T-cell bispecifics (mosunetuzumab and blinatumomab), including the dynamics of B- and T-lymphocytes in circulation, lymphoid tissues, and tumor. The model was developed and validated using mosunetuzumab nonclinical and blinatumomab clinical data. Simulations delineated mechanisms contributing to observed cell and cytokine (IL6) dynamics and predicted that initial step-fractionated dosing limits systemic T-cell activation and cytokine release without compromising tumor response. These results supported a change to a step-fractionated treatment schedule of mosunetuzumab in the ongoing Phase I clinical trial, enabling safer administration of higher doses.

## Introduction

B-cell malignancies constitute a diverse set of diseases, including ~80–85% of non-Hodgkin lymphomas (NHL), and other leukemias such as acute lymphoblastic leukemia (ALL) and chronic lymphocytic leukemia (CLL). B-cell lymphomas and leukemias are biologically heterogenous diseases that are broadly classified as either indolent or aggressive. Indolent diseases such as follicular lymphoma and CLL have a median survival of 8–10 years, whereas aggressive diseases such as diffuse large B-cell lymphoma (DLBCL), and mantle cell lymphoma have a median survival of 6 months if untreated.

Monoclonal antibodies such as rituximab that target the CD20 surface molecule on B-cells, in combination with chemotherapy, have significantly improved the clinical outcome for patients with B-cell malignancies. However, the majority of patients with indolent diseases and about half of patients with aggressive B-cell lymphoma eventually experience relapse or recurrence^1–5^. Recently, T-cell directed therapies including engineered T-cells expressing chimeric antigen receptors (CAR) or bispecific T-cell engager (BiTE) molecules and antibodies have demonstrated efficacy in the treatment of B-cell malignancies. CAR T-cells, such as tisagenlecleucel (Kymriah^®^) and axicabtagene ciloleucel (Yescarta^®^), that target B-cell lineage surface proteins^6,7^ have produced deep and durable responses in patients with relapsed/refractory (r/r) leukemias^8,9^ and lymphomas^10–12^ and have obtained FDA approvals in these indications^13,14^. However, CAR T-cell therapies present a significant risk of severe toxicities, notably cytokine release syndrome (CRS)^15^. In another approach to T-cell based therapy, bispecific molecules simultaneously engage T-cells and tumor cells to stimulate T-cell activation and tumor cell cytolysis. The anti-CD3/CD19 BiTE molecule blinatumomab, a fusion protein of two single-chain antibody fragments, was approved by the FDA in 2014 for treatment of ALL, and clinical response in patients with r/r NHL has been observed^16–19^. Compared with CD19 CAR T-cell therapies, CRS has been less frequent and less severe with blinatumomab treatment, although severe CRS has been observed and remains a potential safety concern^20^. The dose-limiting toxicity for blinatumomab was neurotoxicity, which may also be driven by immune activation but is distinct from CRS^21^; this was mitigated in part by the implementation of a step-up dosing schedule^22^.

Mosunetuzumab is a fully humanized full-length anti-CD20/CD3 T-cell dependent bispecific (TDB) antibody, assembled using a knobs-into-holes technology^23,24^. The mechanism of action of mosunetuzumab is similar to that of blinatumomab: concurrent binding of mosunetuzumab to CD20 on malignant B-cells and CD3 on T-cells leads to T-cell activation and B-cell lysis^25^. Previously, we have shown that mosunetuzumab is highly potent in stimulating T-cell mediated killing of CD20-expressing B-cells, including primary patient-derived leukemia and lymphoma cells both in vitro and in vivo^25^. As mosunetuzumab is a conditional agonist, target B-cell killing is observed only upon simultaneous binding to CD20 on B-cells and CD3 on T-cells. Neither antigen-presentation and co-stimulation nor preexisting T-cell response to tumor is required for activity, and any T-cell, regardless of clonal specificity, activation or differentiation status, can be activated in the presence of mosunetuzumab and CD20^26,27^.

Given the potent T-cell activation induced by mosunetuzumab, toxicities such as the CRS and neurotoxicity observed with blinatumomab and CAR T-cell therapies could impact the therapeutic index of mosunetuzumab in patients. In contrast with blinatumomab, which is administered as a continuous infusion over 4–8 weeks^28^, the pharmacokinetic properties of mosunetuzumab allow for clinical activity with intermittent dosing. These differences in molecular structure, PK, administration, and molecular target prevent extrapolation from blinatumomab clinical experience to mosunetuzumab. Thus, prior to clinical experience with mosunetuzumab, we sought to integrate our preclinical data on mosunetuzumab with clinical data from the empiric optimization of blinatumomab dose schedules to better understand the mechanism of action of mosunetuzumab, support clinical translation, and maximize its therapeutic index in patients with NHL. We developed a mechanistic quantitative systems pharmacology (QSP) model to describe the dynamics of B- and T-lymphocytes and their interactions with the drug in multiple physiological compartments—peripheral blood (PB), tumor, and lymphoid tissues including the spleen, lymph nodes, and bone marrow (BM)—in the presence of either mosunetuzumab or blinatumomab. The model thus captures T-cell activation, cytokine release, and target cell killing to enable concurrent prediction of efficacy- and safety-related biomarkers. This report describes the development of the model and presents model-based evaluation of alternative clinical dosing regimens to reduce the risk of CRS without compromising efficacy, used to support Phase I clinical trial design for mosunetuzumab in NHL (ClinicalTrials.gov ID: NCT02500407).

## Results

### QSP model description

**Fig. 1:**
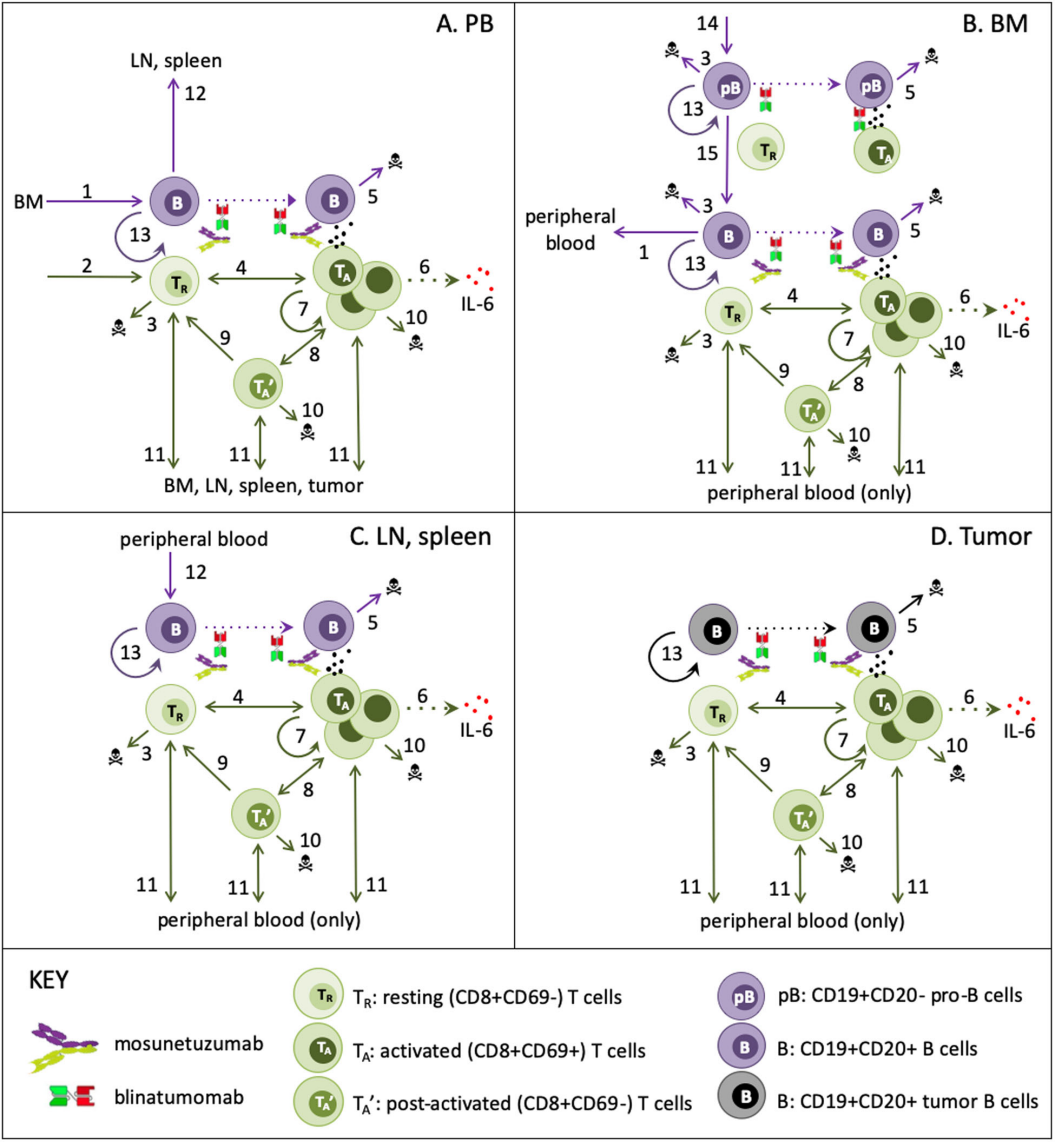
QSP model schematic of T-cell/B-cell bispecifics. The QSP model describes the in vivo dynamics of B and T-cells and their interactions in the presence of mosunetuzumab and blinatumomab interactions in the following compartments: **a** peripheral blood PB, **b** bone marrow BM, **c** separate lymph node and spleen compartments, and **d** an optional NHL tumor compartment. The key biological interactions are labeled in the diagram as follows: (1) migration of newly generated B-cells from BM to PB, (2) Homeostatic thymic generation of new CD8+ T-cells (only when PB T-cells are diminished), (3) homeostatic apoptosis/loss of B-cells (only in BM) and T-cells (only when in excess), (4) T-cell activation due to interaction of bispecific drugs with B and T-cells; for mosunetuzumab, this requires CD20+ B-cells, (5) B-cell death due to T-cell cytolytic activity; for mosunetuzumab, this applies only to CD20+ B-cells, (6) activated T-cell induction of cytokine release, (7) activated T-cell proliferation, (8) interconversion of activated and post-activated T-cells, (9) conversion of post-activated to resting T-cells (e.g., memory cells), (10) activation-related apoptosis of activated and post-activated T-cells, (11) PB T-cell traffic to/from tissues, (12) PB B-cell traffic to replenish normal tissues (only when tissue B-cells are diminished), (13) proliferation of tumor B-cells (constitutive) and normal tissue B-cells (only when diminished), (14) Generation of pro-B-cells from stem cell precursors (15) maturation of CD20- pro-B-cells to CD20+ B-cells. Dotted lines represent mechanisms that do not alter cell states.

We constructed an ordinary-differential-equation based mechanistic model to describe and predict the in vivo dynamics of B- and T-lymphocytes and their interactions in the presence of mosunetuzumab and blinatumomab. The model structure was designed to enable simulation of the biological measurements and processes of interest in a manner consistent with underlying biological mechanism, and preclinical and clinical measurements: specifically, CD8+ T-cell (CD69+ and total) and B-cell numbers; systemic cytokine levels; and tumor growth/regression. The resulting model includes representation of numbers and population dynamics of resting, activated and post-activated CD8+ T-cells and CD19+CD20− and CD19+CD20+ heathy and tumor B-cells as appropriate in PB, lymphoid tissues, and an optional tumor compartment, as illustrated in Fig. 1.

**Fig. 2:**
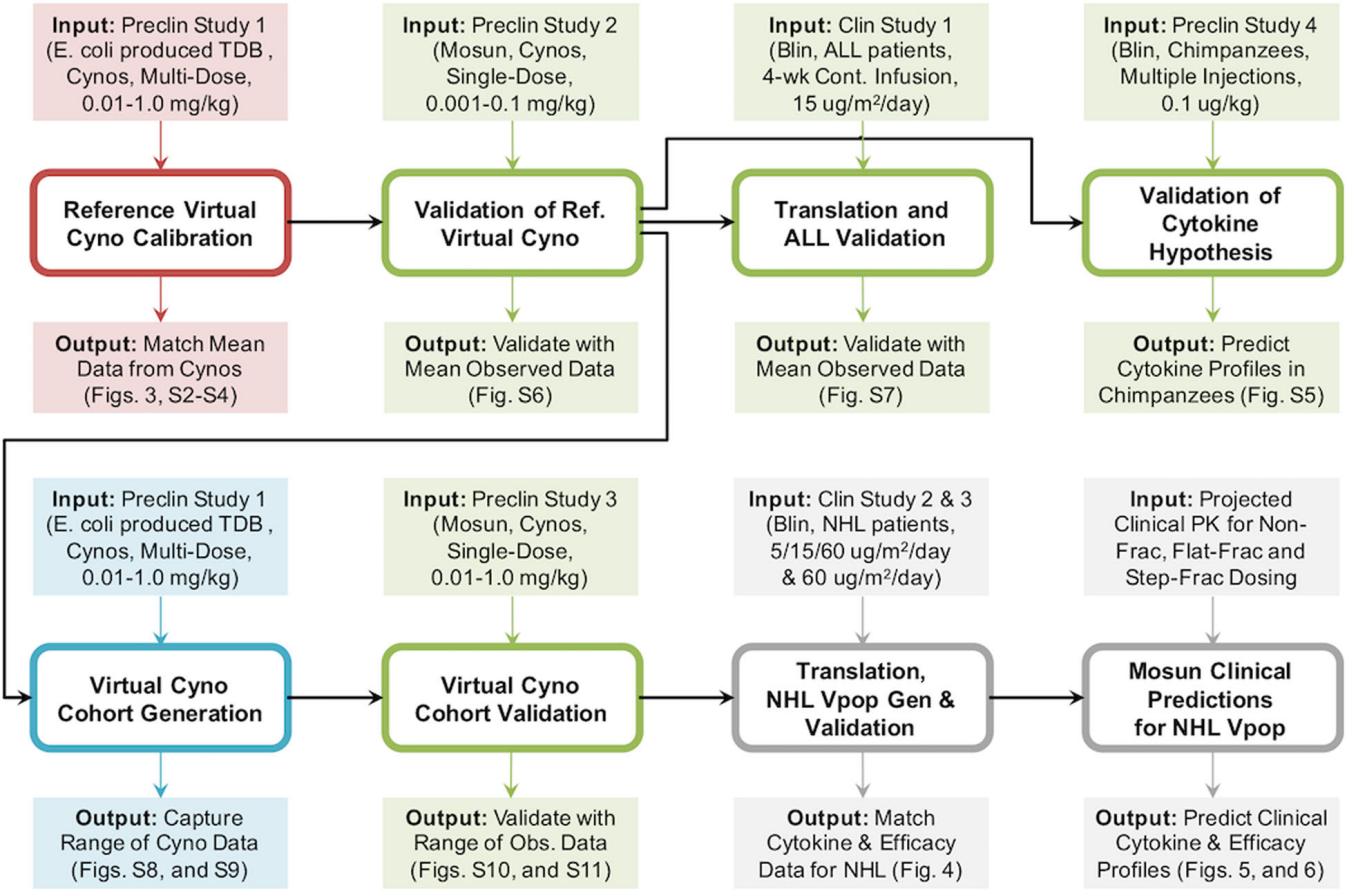
Workflow of mosunetuzumab QSP model development. The QSP model was calibrated using multiple dose preclinical data of E. coli produced anti-CD20/CD3 TDB in cynomolgus monkeys (preclinical study 1, 0.01–1 mg/kg). The outcome was a reference virtual cyno, which reproduces the dynamics of cytokines, B and T-cells in the PB and lymphoid tissues. The model was then validated against data from the single-dose mosunetuzumab in cynomolgus monkeys (preclinical study 2, 0.001–0.1 mg/kg) by using the reference virtual cyno to predict the B and T-cell profiles. Next, the reference virtual cyno was translated to the human ALL patient using the appropriate physiological volume and T and B-cell numbers for different tissues and including blinatumomab PK and its downstream effects on T-cell activation and B-cell killing. The model was successfully validated against the clinical data from blinatumomab in ALL patients. In addition, we used reference virtual cyno and human models to validate the cytokine hypothesis by predicting the IL6 Levels measured in chimpanzees treated with multiple weekly injections of blinatumomab (preclinical study 4). To capture the observed variability in cyno measurements, we generated a virtual cohort of healthy cynos using the range of observed measurements in the multiple dose study of mosunetuzumab in cynomolgus monkeys (preclinical study 1) and validated against the single-dose mosunetuzumab in cynomolgus monkeys (Preclinical study 3, 0.01–1.0 mg/kg). We generated a virtual NHL population by translating the virtual cohort of healthy cynos, adding a tumor compartment by implementing a large B-cell dense mass using human physiology and including additional disease-related variability such as baseline peripheral B and T-cells, tumor load, tumor cell doubling time and revised B:T ratio in the tumor microenvironment. The virtual NHL population matched the distribution of antitumor efficacy and cytokine time profiles following blinatumomab treatment. This population was subsequently used to predict and compare the time course of systemic cytokine levels and antitumor efficacy for different dosing regimens of mosunetuzumab.

Briefly, B-cells and T-cells are represented in the PB, lymphoid tissues (bone marrow—BM, lymph nodes—LN, and spleen), and when appropriate, a lymph-node NHL tumor. Prior to therapy, T-cells are at dynamic equilibrium, with low trafficking rates of resting PB T-cells to/from lymphoid tissues capturing immune surveillance. The bispecific drugs activate T-cells in each compartment, with potency dependent on local drug concentration and B:T-cell ratio, combining effects of drug binding and downstream signaling. In all compartments, activated T-cells proliferate (generating activated and post-activated progeny), kill target-expressing B-cells, induce production of cytokines, and undergo apoptosis or transition to resting or post-activated cells. Post-activated T-cells can undergo apoptosis, convert to resting (e.g., memory) T-cells, or be reactivated. While only activated T-cells can proliferate, all T-cell subsets can traffic from PB to and from other tissues, although activated cells traffic more efficiently/rapidly and have a greater partitioning to peripheral tissues. Exit from the PB of all T-cells is transiently amplified by placebo- or drug-injection stress. Thymic generation of resting PB T-cells and apoptosis of resting T-cells in all tissues restore deviations from homeostatic levels. As mentioned above, activated T-cells can kill target-expressing B-cells in each compartment. B-cell generation and maturation in the BM, unidirectional trafficking from BM to PB and from PB to LN and spleen, and local proliferation in all compartments act to restore diminished B-cell numbers, whereas constitutive proliferation of tumor B-cells captures tumor growth. Greater details on the model implementation and the detailed workflow for generating the results discussed below can be found in Fig. 2 and the “Methods” section.

### Model calibration: a “reference virtual cyno” quantitatively captures and explains response to different dose levels of mosunetuzumab

**Fig. 3:**
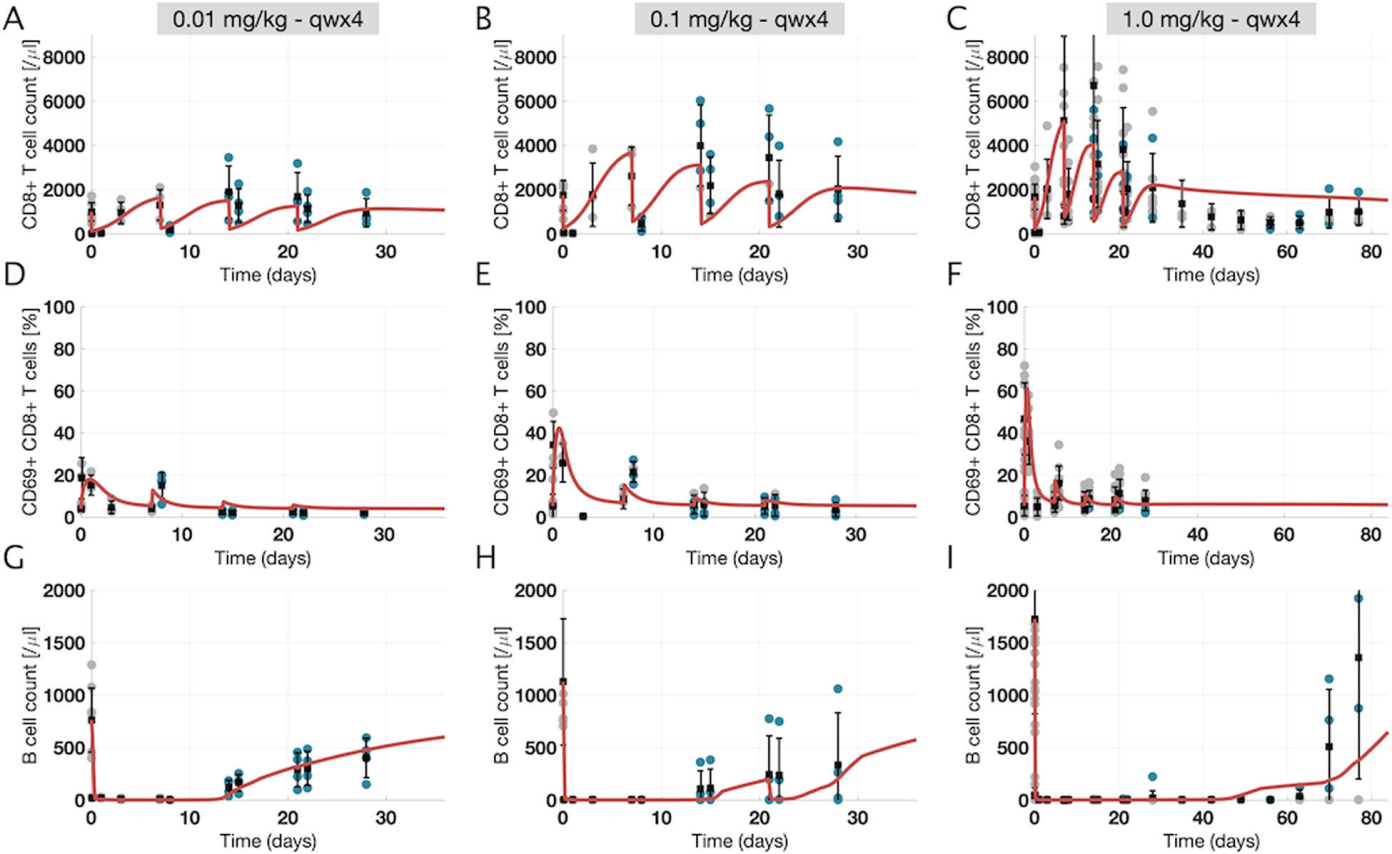
Time profile data and model fits for peripheral blood pharmacodynamic measurements in the multiple dose study of mosunetuzumab in cynomolgus monkeys (preclinical study 1). The reference virtual cyno model replicates T and B-cell dynamics in the peripheral blood for different dose levels of mosunetuzumab in cynos. Each column shows a different dose level, ranging from 0.01 to 1 mg/kg given weekly for 4 weeks. Each row shows a measurement in PB (**a**–**c**: CD8+ T-cells; **d**–**f**: percentage of activated T-cells (CD8+ CD69+ T-cells); and **g–i**: B-cells). Gray and blue dots show individual ADA− and ADA+ data points, respectively (*n* = 4 for 0.01 and 0.1 mg/kg; total *n* = 11 for 1 mg/kg with *n* = 4 after D29 in the recovery phase), and vertical black lines show mean ± standard deviation. Red curves are the model outputs calibrated to data (reference virtual cyno).

The model was calibrated to data from preclinical study 1 (Supplementary Table 1) for multidose treatment of cyno monkeys at three different dose levels. The measured PK was used as an input for the calibration (Supplementary Fig. 1, see “Methods” for details). As shown in the figure, low- and mid-dose but not high-dose groups showed significant antidrug antibodies (ADAs) and loss of drug exposure. Circulating PD measurements are shown in Fig. 3, along with model-derived simulation results (results for control treatment group are provided for comparison in Supplementary Fig. 2). The simulation results generally show good agreement with the preclinical data at all three dose levels, recapitulating the dose-dependent dynamics of PB CD8+ T-cell levels (Fig. 3a–c), percentage of CD8+ CD69+ T-cells (Fig. 3d–f), and B-cell numbers in PB (Fig. 3g–i).

The model further enables us to delineate the mechanisms giving rise to the involved dynamics in the simulations. Upon first exposure to mosunetuzumab, dosing-induced stimulation increases margination and trafficking of all PB T-cells, leading to a rapid drop in circulating T-cells. The posttreatment margination effect has also been observed with other treatments^29–31^. Simultaneously, as the PB T-cells become activated due to drug binding of T- and B-cells, the activated fraction of PB T-cells increases rapidly with higher peaks at higher dose levels, followed by a decline as activated T-cells traffic to tissues. PB T-cell activation leads to rapid depletion of circulating B-cells at all dose levels due to ample drug concentrations and T-cell abundance. T-cells in the tissue are also activated and the activated T-cells proliferate, causing dose-dependent expansion of tissue T-cells which eventually reenter the PB. This process repeats over successive doses, although depletion of circulating target B-cells results in lower PB T-cell activation and the (partial) depletion of tissue B-cells yields progressively less tissue T-cell expansion and reentry into the blood, until T-cells eventually die or revert to an inactive state. PB B-cells eventually recover at around 10 days in the low and medium dose groups due to reduced drug exposure consequent to ADAs (Supplementary Fig. 1), and during posttreatment monitoring in the high-dose group, although the simulated recovery is slower than the average observed recovery (Fig. 3i). Simulated B-cell depletion in lymphoid tissue also capture data from tissue necropsies performed 7 days after the last dose. The very low B:T ratio in the high-dose group indicates sustained depletion in lymph nodes and spleen after the last dose, whereas the higher B:T ratio in the low and mid-dose groups suggests partial B-cell recovery in tissues, which again is consistent with the higher ADA incidence and reduced exposure in these groups (Supplementary Fig. 3).

In addition to cellular dynamics, cytokine levels measured after the first and last dose of mosunetuzumab are also well described by the model (Supplementary Fig. 4). IL6 levels peak 2–6 h after the first dose in a dose-dependent manner and quickly return to baseline levels. IL6 peaks are significantly lower following the last dose of mosunetuzumab relative to the first dose with no obvious dose-dependency observed. IL6 production follows engagement of mosunetuzumab with target T- and B-cells. We assume that activated T-cells either directly or indirectly drive local cytokine production in a manner proportional to their number and activity, i.e., they either produce the IL6 themselves or rapidly stimulate other cells such as monocytes or macrophages to produce IL6^32^. Because T-cell activation occurs in both PB and lymphoid tissues, serum IL6 levels are assumed to reflect contributions from IL6 produced in the PB and IL6 entering the blood from the tissues. After the first dose, when B-cells are present in PB and lymphoid-tissue compartments, the cytokine levels are highest. For the subsequent doses, although activated T-cells are observed in the systemic circulation, ongoing activation of T-cells within the PB is negligible due to circulating B-cell depletion; hence the contribution of IL6 from PB is negligible and most of the measured IL6 is derived from tissue. These data show that the mechanisms incorporated into the mechanistic model for cytokine production are sufficient to characterize cytokine data at different dose levels, and further suggest that for mosunetuzumab, peripheral B-cell depletion can explain the first-dose phenomenon of cytokine release.

Because we use IL6 as an indicator of CRS risk, accurately attributing and capturing its behavior is critical. Thus, to further test the hypothesis that systemic target load can explain cytokine release with B-cell targeted T-cell engagers, we examined data from a study in which chimpanzees were treated with multiple doses of blinatumomab (2 h IV infusion given weekly for 5 weeks). In this study, B-cells were briefly depleted but partially recovered between doses. Animals exhibited recurrent (only slightly reduced) IL6 peaks with each IV administration^33^. For comparison, we simulated similar treatment in the reference virtual cyno and a virtual healthy human (with human tissue volumes and PK) to predict the time profiles of IL6 levels after weekly injections of blinatumomab. In both cases, B-cell depletion was followed by recovery between doses, resulting in repeated cytokine peaks that are qualitatively consistent with the observed cytokine data (Supplementary Fig. 5). Thus, the model captures available preclinical cytokine data.

The model calibration (i.e., the mathematical structure and associated parameter estimates) that successfully describes the circulating and tissue B and T-cells and cytokine levels in cynos in response to mosunetuzumab treatment was designated as the “reference virtual cyno” and used as a starting point for subsequent efforts.

### Validation, translation, and exploration of variability to establish confidence in the model

Having developed the “reference” cyno calibration of the model, we embarked on testing and translational efforts to support model application to clinical use of mosunetuzumab. We verified the model’s ability to predict responses (CD8+ T-cell numbers, CD8+ activated fraction, and B-cell numbers) of cynos to single dose of mosunetuzumab. This provided confidence in the ability of the model to predict on-treatment lymphocyte dynamics in response to an alternate dosing regimen (single dose) and a wider range of doses to as low as 0.001 mg/kg (Supplementary Fig. 6).

We then tested the translational relevance of the model by prediction of published clinical data from blinatumomab treatment of ALL patients. Scaling the cyno parameters based on known physiological differences (tissue volumes, cell numbers) between cynos and humans and baseline data on ALL patients, we successfully predicted the patients’ reported peripheral T- and B-cell response to blinatumomab (Supplementary Fig. 7). This provided confidence in the utility of the model for predicting clinical responses to T-cell engaging agents in patients with B-cell malignancies. Finally, in order to address intersubject variability as well as the biological and parameter uncertainty, we generated a virtual cohort of healthy cynos to capture the observed variability in the multidose cyno study, using a random search method to generate alternate parameters locally randomized around the initial fits and within reasonable parameter ranges (Supplementary Figs. 8, 9; see Supplementary Method 2 for details). We then confirmed that the virtual cynos successfully predicted the ranges in the PB and lymphoid-tissue measurements across the dose levels in the single-dose cyno study (Supplementary Figs. 10, 11). This established confidence in our ability to expand from one reference subject to a virtual cohort that can capture the variability of response across a wide dose range. The success of the validation, translation, and cohort simulation efforts above provided the required confidence to proceed with application to NHL predictions.

### A virtual population of NHL patients reproduces the antitumor efficacy and systemic cytokine data observed in blinatumomab-treated patients

**Fig. 4:**
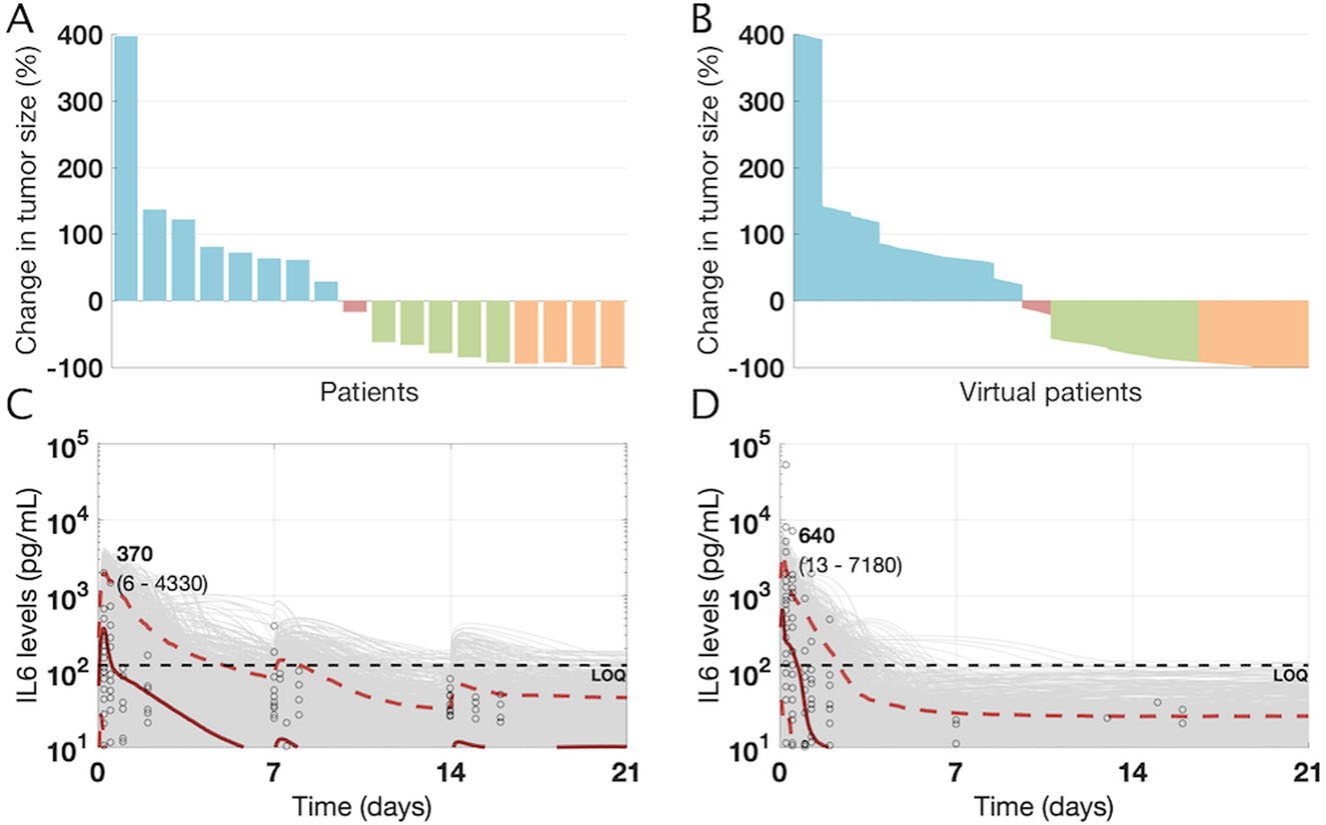
Clinical data and model outputs for antitumor efficacy and cytokine profiles in DLBCL/NHL patients following treatment with blinatumomab. **a** Efficacy data for patients (*n* = 18) treated with continuous infusion of blinatumomab for 8 weeks using a step-up dosing regimen is shown^19^. **b** The overall antitumor efficacy from virtual population of NHL patients (*n* = 4500 virtual patients) treated with Blinatumomab match the observed clinical data. **c**, **d** The cytokine (IL6) levels from^34^ are digitized and replotted for two cohorts of patients treated with either continuous infusion of blinatumomab using (**c**) a step-up dose with 5 μg/m^2^/day for the 1st week, 15 μg/m^2^/day for the 2nd week and 60 μg/m^2^/day for the 3rd week or (**d**) continuous infusion of blinatumomab at 60 μg/m^2^/day for 3 weeks. The cytokine characteristics of the virtual population of NHL patients was calibrated using the step-up dosing schedule and subsequently validated against the cytokine levels for blinatumomab treatment at 60 μg/m^2^/day. Black dots show digitized cytokine data (*n* = 21 for 5/15/60 μg/m^2^/day; *n* = 9 for 60 μg/m^2^/day), gray curves represent individual virtual patients and the red curves represent median, 5, and 95 percentiles for cytokine profiles across the virtual population, consistent with the median and range of measured cytokines. Numbers shown in bold indicate median IL6 peaks whereas numbers shown in parentheses indicate the 5–95 percentile range. Note that it was not technically feasible to digitally capture all the cytokine data/levels that were overlaid at the horizontal line corresponding to 10 pg/mL.

In order to model DLBCL, we modified the virtual cyno cohort described above by translating physiological parameters to human values and including a tumor compartment with additional variability in tumor parameters. We used the resulting NHL virtual patients to create a virtual NHL population whose antitumor response to simulated therapy matched the data observed in relapsed/refractory (r/r) DLBCL patients treated with an 8-week continuous infusion of blinatumomab using a step-up dosing regimen (data obtained from^19^ and shown in Fig. 4a; see “Methods” for details). Figure 4b shows the waterfall plot of tumor growth/regression in the virtual population of DLBCL patients matches the observed data, with a wide range of outcomes from progressive disease to complete response (CR).

The same virtual population was also used to reproduce cytokine (IL6) dynamics observed in NHL patients receiving continuous infusion of blinatumomab in either a step-up dosing regimen (5 μg/m^2^/day for the 1st week, 15 μg/m^2^/day for the 2nd week, 60 μg/m^2^/day for the 3rd week) or a constant regimen of blinatumomab (60 μg/m^2^/day throughout)^34^. IL6 data from the step-up dosing schedule were used to scale the IL6 production rate constant ($k_{prod}^{IL6}$) and the relative fractional contribution of tissue cytokines ($f_{tiss}^{IL6}$ < 1) to quantitatively match the measured serum cytokine levels (see “Methods” for details of cytokine implementation in the model).

As seen in Fig. 4c, simulations in the NHL virtual population recapitulate the clinical cytokine data for the step-up regimen, with peak cytokine levels attained within 24 h and declining within 2 days of the start of infusion. Furthermore, simulation of the constant regimen in the NHL virtual population successfully predicted the cytokine levels for blinatumomab on this dosing schedule (Fig. 4d), capturing the higher peaks not seen in the step-up regimen. As indicated by the data and recapitulated by the model, the first cytokine peak is dose dependent, and the subsequent peaks are roughly an order of magnitude lower. As in the preclinical exploration, the model suggests that first IL6 peak is primarily driven by production in the PB and the contribution of tissue cytokines to serum cytokine levels is minimal, whereas the subsequent peaks primarily reflect the contribution of tissue/tumor IL6 after PB but not tumor B-cells are depleted. Thus, the virtual NHL population satisfactorily captures and predicts clinical data on both the safety surrogate biomarker (IL6 levels) and efficacy (tumor B-cell killing) for DLBCL and is suitable for predicting the results for mosunetuzumab in a similar patient population.

### Step fractionation of mosunetuzumab is predicted to reduce peak systemic cytokine release and T-cells activation without compromising tumor cell killing

Using the virtual NHL population, we simulated the effect of 4 cycles (21 days each) of mosunetuzumab treatment on serum cytokine kinetics, activated T-cells dynamics, and the antitumor efficacy at Day 84, to compare the following dosing regimens: (1) a non-fractionated dosing schedule, in which patients receive a single dose of 20 mg administered every 21 days (on Day 1 of each cycle); (2) a flat-fractionated dosing schedule in Cycle 1, in which patients receive a dose of 6.7 mg on Day 1, Day 8, and Day 15 of Cycle 1, followed by a dose of 20 mg on Day 1 of subsequent cycles; (3) a double-step fractionated dosing schedule in Cycle 1, in which patients are administered doses of 1.6, 10, and 10 mg on Day 1, Day 8, and Day 15 of Cycle 1, respectively, followed by administration of 20 mg on Day 1 of subsequent cycles; and (4) a single-step fractionated dosing schedule in Cycle 1, in which patients are administered doses of 1.6, and 20 mg on Day 1, and Day 8 of Cycle 1, respectively, followed by administration of 20 mg on Day 1 of subsequent cycles. All simulations were performed using the linear PK model for mosunetuzumab.

**Fig. 5:**
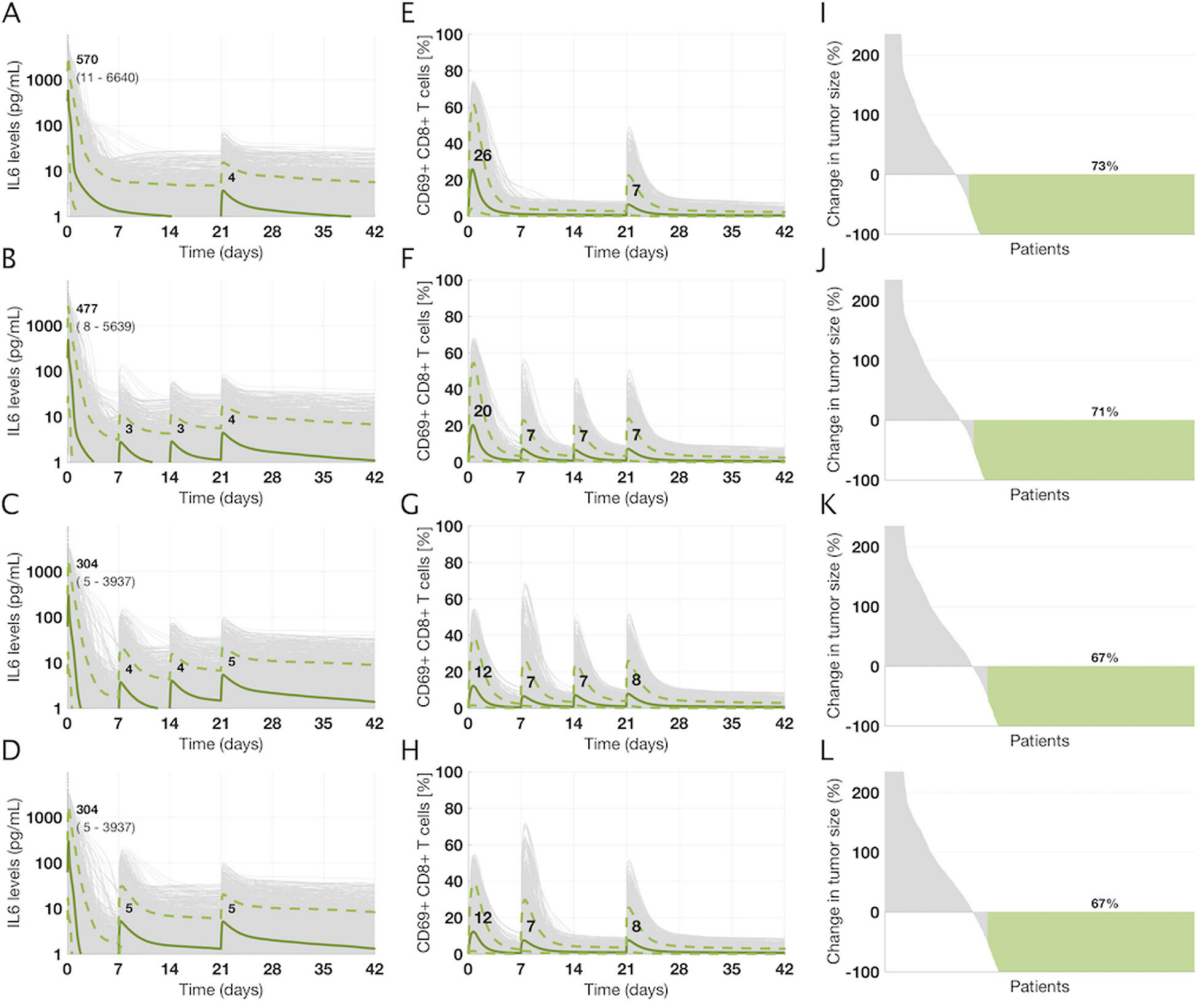
Projected time profiles of cytokines, activated T-cells, and antitumor efficacy in NHL patients treated with different dosing regimens of mosunetuzumab. The NHL virtual population was used to predict and compare IL6 levels, fraction of activated T-cells and efficacy profiles for different dosing regimens (**a**, **e**, and **i**: 20 mg on Cycle 1 Day 1 (C1D1); **b**, **f**, and **j**: 6.7 mg on C1D1, C1D8, C1D15; **c**, **g**, and **k**: 1.6/10/10 mg on C1D1, C1D8, and C1D15; **d**, **h**, and **l**: 1.6/20 mg on C1D1, and C1D8; in all regimens 20 mg is administered on Day 1 of subsequent cycles). Gray curves represent individual virtual patients and green curves represent median, 5, and 95 percentiles for cytokine and activated T-cell profiles across the virtual population. Numbers shown in bold indicate median IL6 peaks whereas numbers shown in parentheses indicate the 5–95 percentile range. In the third column, the numbers indicate percentage of virtual patients with more than 50% reduction in tumor size on Day 84 and are only used for comparison purposes across dosing regimens.

The model predictions for IL6 levels suggest that, as observed for blinatumomab, peak cytokine levels are driven primarily by the Cycle 1 Day 1 dose and that a higher dose on Cycle 1 Day 1 leads to higher peak cytokine levels (Fig. 5a–d); thus, the first cytokine peak for the non-fractionated dosing schedule is higher than that of the flat-fractionated dosing schedule, which in turn is higher than that of the step-fractionated dosing schedule. In addition, the model suggests that for the single- or double-step fractionated schedule, subsequent cytokine peaks are substantially lower than the first cytokine peak despite administration of a higher dose of mosunetuzumab, providing the opportunity to escalate to higher doses. Comparing the single- and double-step fractionation suggests that the additional fractionated dose does not mitigate cytokine peaks beyond C1D1, hence providing the opportunity to escalate to the target dose as early as Day 8 in Cycle 1.

With respect to the proportion of systemic activated T-cells arising from mosunetuzumab treatment (Fig. 5e–h), the model suggests that the first spike in Cycle 1 is dose-dependent (non-fractionated > flat-fractionated > step-fractionated dosing) and the subsequent spikes after each dose regardless of the time of administration are generally similar, suggesting that further fractionation has minimal impact on T-cell activation peaks.

Importantly, to evaluate whether flat or step-up dose fractionation would significantly reduce antitumor efficacy, we compared the waterfall plots and found that percent change in tumor size on Day 84 is overall similar across the dosing regimens, despite the slight delay in reaching maximum doses and the relatively rapid growth of DLBCL tumors (Fig. 5i–l). Hence, the model predicts that step-fractionated dosing regimens mitigate the risk of high systemic cytokine peaks across the population of NHL patients, with minimal impact on antitumor efficacy, suggesting that this would be a safer option for administration of mosunetuzumab to patients.

### Preliminary clinical results match prospective model predictions

**Fig. 6:**
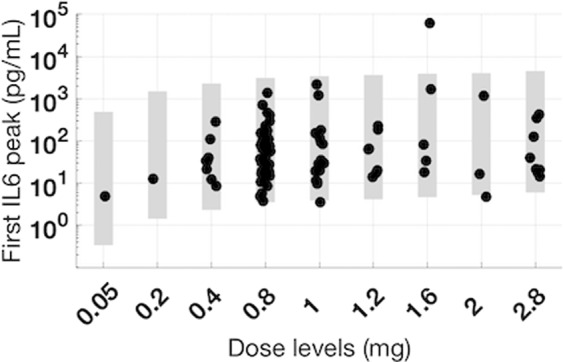
Projected range of first IL6 peaks post mosunetuzumab treatment in NHL patients and comparison with clinical data. The NHL virtual population was used to project range of clinical IL6 peaks after the first dose of Cycle 1 for a range of clinically tested doses of mosunetuzumab (0.05–2.8 mg). The gray shaded region represents the range of simulated IL6 peak after the first dose of mosunetuzumab was administered. The black dots represent the first IL6 peak in mosunetuzumab Phase 1 clinical data (*n* = 1, 2, 8, 47, 17, 6, 5, 3, 8 for 0.05, 0.2, 0.4, 0.8, 1, 1.2, 1.6, 2, 2.8 mg, respectively).

In a Phase 1 clinical trial, we have tested a range of mosunetuzumab doses from 0.05 to 2.8 mg for the Cycle 1 Day 1 dose in patients with r/r NHL. Overall, we observed that cycle 1 double-step fractionated dosing of mosunetuzumab appears to mitigate CRS-related toxicity in r/r NHL patients. In addition, maximum levels of peripheral T-cell activation and IL6 elevation occur after the first dose of Cycle 1^35^. Both of these are consistent with the model predictions. Further quantitative validation is provided by comparison of available first-dose peak IL6 data with model predictions, shown in Fig. 6. Simulations accurately predicted the levels and ranges of peak IL6 levels measured in more than 95 patients in our Phase 1 clinical trial, with only one exception in cohort 1.6 mg for a patient whose IL6 levels exceeded the range predicted by the model; however, this patient is also an outlier with respect to the clinical data, with IL6 levels about 30-fold higher than the next highest measurement. Thus, the increased safety with the altered clinical strategy and the emerging cytokine data support the accuracy and value of the model predictions. This toxicity-mitigation dosing strategy has enabled administration of higher, more efficacious doses without reaching maximum tolerated dose^35^.

## Discussion

In this work, we developed and used a mechanistic model of B-cell targeted T-cell engaging bispecific drugs to support the translation of preclinical experience with the CD20/CD3 bispecific antibody, mosunetuzumab, to clinical predictions on the relative safety and efficacy of different dose regimens in NHL. The model describes the dynamics of B and T-lymphocytes and their interactions in multiple physiological compartments in the presence of mosunetuzumab or the CD19-targeting BiTE blinatumomab. The model was built and calibrated using in vitro potency data and pharmacodynamic data in cynomolgus monkeys treated with mosunetuzumab. Preclinical and translational validation were performed using additional mosunetuzumab preclinical data and blinatumomab clinical data in r/r ALL, providing confidence in the predictive capabilities of the model for different B-cell targets (CD19 vs. CD20), species (cyno vs. human), drug format and PK (BiTE vs. full-length antibody), and B-cell malignancies (ALL and DLBCL). The dynamics of activated CD8+ T-cells, total CD8+ T-cells, and target cells in circulation and tissues were well-characterized by the model for a wide range of mosunetuzumab doses from 0.001 to 1 mg/kg, which encompasses the projected range of doses currently being tested in a first-in-human Phase I study. The model was used to inform the clinical design by evaluating the proposed dosing strategies to maximize the therapeutic index of mosunetuzumab in patients with r/r NHL, based on prior clinical experience with blinatumomab as well as adoptive T-cell therapies (e.g., CAR T-cells) for the treatment of hematologic malignancies. The results suggested that a single-step or double-step fractionated dosing would mitigate peak cytokine levels with minimal impact on antitumor response. This result provided a strong rationale and quantitative guidance for the dosing schedule in the Phase I clinical trial of mosunetuzumab (ClinicalTrials.gov Identifier NCT02500407). Emerging results from the trial appear to confirm the predictions and have enabled higher dosing with reduced safety events.

Although clinical data on step-up dosing with blinatumomab provided some insight on how to reduce first cytokine peak, we could not directly extrapolate from blinatumomab experience due to the fundamental differences between blinatumomab and mosunetuzumab in structural format and PK (short half-life of two linked single-chain antibody fragments vs. longer half-life of a full-length antibody), administration (continuous vs. intermittent dosing), and potency (different EC50 values for T-cell activation and B-cell killing). In addition, the model enabled us to explore whether there would be a detrimental effect of step-fractionated dosing on efficacy, whereas data were not available comparing efficacy for step-up vs. constant dose regimens of blinatumomab. This was an important consideration, especially for the treatment of aggressive tumors such as DLBCL.

A range of different models for T-cell engaging agents have been presented in recent years. These are typically based on preclinical data and include consideration of factors such as target levels and binding interactions along with PK to explore how drug properties considerations can influence tissue distribution and efficacy^36^, to capture and predict preclinical responses for different dose regimens^37^, to propose first-in-human dose^38^, and to project efficacious dose^39^ for various different bispecific agents. Each model is tailored to its application and has unique strengths. To our knowledge, ours is the first to explicitly include both blood and lymphoid tissues, address preclinical and clinical settings, include both safety (cytokines) and efficacy (target cell depletion) predictions, and explicitly address population variability. In our work, we have focused on including both efficacy and safety readouts to make more robust predictions to inform dosing strategies in highly variable clinical populations, whereas the prior studies have focused on cell killing and efficacy and not safety readouts that can limit dose levels.

Predicting the impact of dosing regimen on clinical IL6 levels, as a surrogate biomarker for CRS, was a primary goal in this application of the model. While there is clear evidence for the central role of IL6 in CRS^40,41^, neither has a clear correlation between IL6 levels and severity of CRS been established, nor is there an accepted IL6 threshold above which patients will experience CRS. Nevertheless, higher IL6 levels are generally associated with a higher risk of severe CRS, and anti-IL6R antibody tocilizumab has been approved for the treatment of severe and life-threatening CRS arising following administration of CAR T-cell therapies^42^. Thus, IL6 serves as a reasonable surrogate biomarker for CRS risk, and our goal in comparing different dosing strategies was to identify a dosing regimen that would reduce IL6 levels across the patient population.

Preclinical studies with mosunetuzumab and blinatumomab and clinical observations with blinatumomab have both shown peak release of IL6 and other cytokines upon first administration of drug, followed by drastically reduced cytokine release upon subsequent doses^34^. These observations might reflect desensitization of cytokine-producing cells. However, we found our model could capture the time-dependent cytokine attenuation observed for both blinatumomab and mosunetuzumab in nonhuman primates and human B-cell malignancies without invoking immune desensitization or similar regulatory mechanisms. We implemented only the established mechanism of target- and drug-dependent activation of T-cells in each compartment and represented cytokine production as a consequence of this activation. We did not explicitly represent different cellular sources of IL6, and instead assumed that activated T-cells through direct or indirect mechanisms drive cytokine production, either producing the IL6 themselves or rapidly stimulating other cells such as macrophages to produce IL6 in a manner proportional to activated T-cell numbers and activation signals^32^. With this implementation, we find that if initial administration of blinatumomab or mosunetuzumab is sufficient to deplete peripheral B-cells, systemic T-cell activation and cytokine production upon subsequent drug administration is greatly attenuated. In fact, recurrent cytokine peaks observed in blinatumomab-treated chimpanzees with partial B-cell recovery suggests that cytokine release need not be a first dose phenomenon and that systemic target abundance may play a role. However, this does not exclude a potential role for immune desensitization or other mechanisms such as exhaustion of cytokine-producing capability, as drivers of cytokine attenuation, and normal and tumor B-cells may differentially influence the PD consequences of T/B-cell engaging agents such as mosunetuzumab and blinatumomab. Clinical studies in newly diagnosed DLBCL patients who have not been exposed to prior treatments and are not B-cell depleted offer an important opportunity to distinguish between normal and tumor B-cell effects and determine how strongly circulating B-cell counts influence cytokine release. Finally, the relative roles of systemic target depletion and other mechanisms influencing cytokine secretion might differ significantly among indications, and especially between hematological malignancies and solid tumors.

We have compared the model predicted range of IL6 peaks after the first dose with the clinical data. The virtual population used in this work explores the variability in the IL6 module and hence we have quantitative confidence in the predictions for IL6. However, limited variability was explored for the efficacy-related parameters due to the limited patient data that we had from blinatumomab study. Since the focus was comparison of dosing regimens, we have confidence on the relative efficacy outputs comparing across the dosing regimens. Additional clinical data would be required to quantitatively validate the predicted tumor regression rates.

In the model, we have focused primarily on PD, with a fit-for-purpose approach to PK. Rather than using a PK model for calibration of the virtual cyno, we used the PK measured in cyno studies directly, allowing us to exactly capture each animal’s PK, including the effect of ADAs. ADAs were observed broadly in low- and mid- but not high-dose animal groups. This pattern of dose-dependent ADA effect is frequently attributed to high drug concentrations overcoming the impact of ADA on PK and to ADA assay interference at high drug concentrations. It is however possible that the more thorough depletion of B-cells is responsible for the reduced ADA in the high-dose group. Even so, this should not be a serious concern for the use of low doses in the step-up regimen, because the step-up phase is limited to the first 2 weeks (Days 0–14) during which ADA responses are not yet fully developed. Furthermore, preclinical ADAs are not predictive of clinical ADAs. Thus, our clinical predictions assume no significant ADA impact on PK; for blinatumomab, reported immunogenicity rates are <1%^43^, and analysis of mosunetuzumab clinical data collected so far has shown no evidence of ADA^35^.

We also used a simplified representation of pharmacokinetics (a linear two-compartment model) for prospective simulation in human. We acknowledge that the preclinical PK is nonlinear as demonstrated by the population PK model in^44^, however, in the absence of clinical PK data, the linear PK model was used for the purpose of IL6 predictions, as cytokine levels typically peak and drop within 24–48 h, for which the projected PK profiles from the linear and nonlinear PK models are comparable. Future work can include the PK data from clinical studies. Mechanistic representations of the effects of target levels, turnover, and binding affinity can also help capture nonlinear PK and downstream PD. Such mechanisms have been included in previous preclinical modeling efforts^36–39^. However, this requires either knowledge or calibration of expression and turnover of all targets (CD3, CD19, CD20, including subsequent alteration due to drug action) in all tissues, species, and conditions modeled, which becomes more challenging in clinical application, especially in peripheral tissues for which clinical measurements are not available. Thus, we have used the linear PK with approximate partition coefficient-based distribution to tissues, and we have modeled the direct drug effects as a function of T:B ratio and drug concentration, based on corresponding in vitro data for each drug to implicitly account for the ternary-synapse formation and downstream cellular function. The reasonable behavior and validation of the model over a broad range of drug concentration and T:B ratios supports this approach.

Beyond the specific application to the candidate dose regimen assessment presented, the model serves to integrate preclinical and clinical biomarker data from related molecules and indications in a unified quantitative explanation of the biology of T-cell engaging agents in B-cell malignancies. This single mathematical description of the mechanisms of drug and target cell-dependent T-cell activation, proliferation, margination/migration, cytokine secretion, cytotoxicity, and target cell depletion quantitatively describes and predicts the diverse blood and tissue biomarker data for the two species (cyno and human), two drug molecules with similar mechanisms of action but very different PK properties (blinatumomab and mosunetuzumab), and three pathological/physiological contexts (healthy cynos, human ALL, and human NHL) considered. This broad fidelity increases our confidence in our understanding of the mechanisms triggered by T-cell recruiting bispecifics. The consistency of the systemic cytokines with systemic target load and drug level for example improves our understanding of mechanisms driving toxicity, whereas various other factors such as tumor proliferation rates and the abundance of T-cells relative to tumor cells influence efficacy.

Overall, the systems modeling presented here offered a novel and valuable approach for evaluation of clinical dosing strategies for mosunetuzumab and can potentially be extended to other related molecules and/or other B-cell malignancies. Potential future applications of the systems model would be to inform the clinical efficacious dose projections and combination strategies of mosunetuzumab with other therapeutics to achieve a more favorable benefit-risk profile in the patient population of interest.

## Methods

### Datasets used in the workflow of QSP model development

Preclinical and clinical measurements were either obtained from published literature or directly from studies conducted by Genentech. A summary of all datasets and where each of which was used in the workflow is shown in Supplementary Table 1. Note that in preclinical study 1, an E. coli produced anti-CD20/CD3 TDB was used, whereas in preclinical studies 2 and 3, mosunetuzumab was used. A bridging study conducted by Genentech suggested that the pharmacokinetics and pharmacodynamics of these agents are similar. Hence, in this work we do not differentiate between the molecules and we use mosunetuzumab in the rest of this paper for consistency.

### PK data and modeling

In model development, calibration, and validation, we used measured PK data as a specified input (forcing function) to the QSP model. In the multiple dose preclinical study 1 (Supplementary Table 1), mosunetuzumab serum concentrations showed apparent dose-proportional clearance from Day 0 to 7 in cyno, but starting with the second dose, rapid clearance was observed (4 out of 4 at 0.01, 2 out of 4 at 0.1, and 1 out of 4 at 1 mg/kg) corresponding to ADA generation (Supplementary Fig. 1). PK profiles at the higher doses suggested time-dependent clearance; however, data were comparably well-fit assuming linear clearance. Thus, for clinical predictions, we used a linear two-compartment PK model with allometric scaling of CL to project clinical PK profiles.

### QSP model structure

As depicted in Fig. 1, the model structure includes the following components and mechanisms:

1. Five physiological compartments: PB, lymphoid tissues including the spleen, lymph nodes, and BM, and an optional lymphoid-tissue embedded tumor used to model NHL only.
2. Three subsets of CD8+ T-lymphocytes (resting, activated, and post activated) in the aforementioned compartments and their population dynamics; specifically: thymic generation of resting cells or apoptosis of resting cells when T-cells are respectively below or in excess of homeostatic numbers; activation of resting and post-activated cells (see below); proliferation of activated T-cells resulting in activated and post-activated daughter cells; deactivation of activated cells to resting or post-activated cells; activation-related apoptosis of activated and post-activated cells, and inter-compartment trafficking of all subsets described below.
3. A T-cell activation signal dependent on drug concentration and cell abundance, based on a Michaelis–Menten formulation for activation as a function of drug concentration with a Vmax dependent on B:T ratio. (both relationships are drug specific and independently specified for blinatumomab and mosunetuzumab).
4. Trafficking of T-cells between PB and any of the other tissues (BM, spleen, LN, tumor), with more rapid trafficking and greater tissue partitioning of activated T-cells compared to resting or post-activated cells.
5. Two subpopulation of B-cells, CD19+CD20− (pro-B, denoted as “pB”) and CD19+CD20+ (pre- to mature-B, denoted as “B”), in the BM; only the CD19+CD20+ B-cell subset is represented in other tissues.
6. Unidirectional trafficking of CD19+CD20+ B-cells from BM to PB and from PB to other tissues (spleen and LN) when B-cell numbers in the target compartment(s) are below homeostatic levels.”
7. Additional population dynamics of B-cells (specifically, generation, maturation, and apoptosis in the BM; cytotoxic death in all compartments; and when normal B-cell numbers are in deficit, local proliferation in all normal tissues, and traffic of PB B-cells to other compartments), and pathophysiological alteration thereof (altered baseline B- and T-cells in PB and tissues for ALL and addition of a tumor with B-cell proliferation for NHL).
8. Pharmacokinetics of mosunetuzumab and blinatumomab and their concentration- and cell-composition dependent activation of CD8+ T-lymphocytes and consequent cytokine production (see below) and killing of CD20+ and CD19+ B-lymphocytes, respectively.
9. Cytokine production (induced) by activated T-cells, dependent on instantaneous local cytokine production signal, again determined by drug concentration and decay in the number of target cells.
10. Healthy and disease physiology for B- and T-cells in different tissues (DLBCL: large B-cell dense LN tumor; revised T/B-cell numbers and turnover).

The model was developed in SimBiology (MathWorks, Natick, MA), in which tissue compartments 1, 2, and 3 correspond to spleen, lymph nodes, and BM, respectively. See Supplementary Method 1 for detailed description of the QSP model and Supplementary Table 2 for parameters used in the model.

Due to the mechanistic complexity of the model relative to the available data, the model is underspecified and parameter values are not identifiable, as is often the case with systems biology/pharmacology models. However, such models typically include many so-called “sloppy” parameters, that may not impact behaviors of interest. Rather, the use of such models is focused on the range of behaviors and predictions and the subset of crucial parameters that influence them, rather than precise parameter identification. Thus, in this work, we employ previously established virtual cohort and population methodologies^40,45^ for exploration of uncertainty and intersubject variability in crucial parameters and their influence on behaviors of interest. Prior work has established these approaches as enabling accurate predictions with underspecified models^40,46^. The workflow of sequential steps for calibration and application of the QSP model across multiple independently conducted in vivo studies of single and multiple dose mosunetuzumab is shown in Fig. 2, starting with model calibration and leading up through clinical predictions. For each step, the data used as input and the corresponding outcome are also shown to explain how different sources of data were integrated in the model and what they enabled. Greater detail is provided in Supplementary Method 2.

## Supplementary information

pdf version of Supplementary Document

Supplementary Table 2

Supplementary Data File 1

## Data Availability

The datasets generated and/or analyzed during the current study are available from the corresponding author on reasonable request.

## Code availability

The code for execution of the model is available in the Supplementary Materials.
