# Supplementary material for: Mitigating the risk of cytokine release syndrome in a Phase I trial of CD20/CD3 bispecific antibody mosunetuzumab in NHL: impact of translational system modeling
Source: NPJ Syst Biol Appl. 2020 Aug 28;6:28. doi: 10.1038/s41540-020-00145-7 (PMC7455723; doi:10.1038/s41540-020-00145-7)
Supplement: Supplementary file 1 — pdf version of Supplementary Document [file 41540_2020_145_MOESM1_ESM.pdf]

Title: Mitigating the Risk of Cytokine Release Syndrome in a Phase I Trial of CD20/CD3 Bispecific Antibody Mosunetuzumab in NHL: Impact of Translational System Modeling

Authors: Iraj Hosseini[1], Kapil Gadkar [1], Eric Stefanich[1], Chi-Chung Li[1], Liping L. Sun[1], Yu-Waye Chu[1], and Saroja Ramanujan[1]*

Affiliations:

[1]Genentech Inc., South San Francisco, CA.

*To whom correspondence should be addressed: ramanujan.saroja@gene.com.

Running title: Translational Systems Model of CD20/CD3 Bispecific Antibody

Keywords: Systems Pharmacology, Cytokine Release Syndrome, IL6, Non-Hodgkin Lymphoma, T-cell dependent Bispecific Antibody

Abbreviations: ADA – anti-drug antibodies; ALL – acute lymphoblastic leukemia; BiTE – bispecific T-cell engager; BM – bone marrow; CAR – chimeric antigen receptors; CLL – chronic lymphocytic leukemia; CR – complete response; CRS – cytokine release syndrome; DLBCL – diffuse large B-cell lymphoma; FL – follicular lymphoma; LN – lymph node(s); MCL – mantle cell lymphoma; MTD – maximum tolerated dose; NHL – non-Hodgkin lymphoma; PB – peripheral blood; PD – pharmacodynamic(s); PK – pharmacokinetic(s); QSP – quantitative systems pharmacology; r/r – relapsed/refractory; SP – spleen; TDB – T-cell dependent bispecific

Supplementary Materials:

Supplementary Method 1. Detailed Description of the QSP Model

CD8+ T-cell dynamics

In the model, the total population of CD8+ T-cells consists of three subpopulations: 1) resting T-cells, which can be thought of as the entire population of CD8+ T-cells at homeostasis in the absence of the drug; this population can be activated upon exposure to mosunetuzumab or blinatumomab in the presence of the target cells; 2) activated T-cells, which are cells that have been activated by mosunetuzumab, express CD69, undergo proliferation and can kill the target cells; and 3) post-activated, de-activated, or exhausted T-cells, which are cells that are in transition from the active state to a resting state or death; these T-cells no longer express CD69, cannot proliferate, secrete cytokine, or kill target cells and unless they are re-activated, will either die or revert to resting cells. Note that the subpopulation of post-activated T-cells is included in the model in order to reproduce the observed T-cell data in our preclinical studies. The following equations govern the dynamics of T-cell sub-populations in the model.

Dynamics of resting CD8+ T-cells in peripheral blood:

$$\frac{dT_r^{PB}}{dt} = \text{Gen} - \text{Apop} - \text{Act}\,(T_r^{PB} \leftrightarrow T_a^{PB}) + \text{Deact}(T_{a'}^{PB} \rightarrow T_r^{PB})$$

$$-\text{Traffic to SP}(T_r^{PB} \leftrightarrow T_r^{SP}) - \text{Traffic to LNs}(T_r^{PB} \leftrightarrow T_r^{LN})$$

$$-\text{Traffic to BM}(T_r^{PB} \leftrightarrow T_r^{BM}) - \text{Traffic to Tumor}(T_r^{PB} \leftrightarrow T_r^{Tumor}),$$

Dynamics of activated CD8+ T-cells in peripheral blood:

$$\frac{dT_a^{PB}}{dt} = \text{Act}\,(T_r^{PB} \leftrightarrow T_a^{PB}) + \text{Act}\,(T_{a'}^{PB} \leftrightarrow T_a^{PB}) - \text{Apop}$$

$$-\text{Traffic to SP}(T_a^{PB} \leftrightarrow T_a^{SP}) - \text{Traffic to LNs}(T_a^{PB} \leftrightarrow T_a^{LN})$$

$$-\text{Traffic to BM}(T_a^{PB} \leftrightarrow T_a^{BM}) - \text{Traffic to Tumor}(T_a^{PB} \leftrightarrow T_a^{Tumor}),$$

Dynamics of post-activated CD8+ T-cells in peripheral blood:

$$\frac{dT_{a'}^{PB}}{dt} = -\text{Deact}(T_{a'}^{PB} \rightarrow T_r^{PB}) - \text{Act}\,(T_{a'}^{PB} \leftrightarrow T_a^{PB}) - \text{Apop} + \text{Prolif}$$

$$-\text{Traffic to SP}(T_{a'}^{PB} \leftrightarrow T_{a'}^{SP}) - \text{Traffic to LNs}(T_{a'}^{PB} \leftrightarrow T_{a'}^{LN})$$

$$-\text{Traffic to BM}(T_{a'}^{PB} \leftrightarrow T_{a'}^{BM}) - \text{Traffic to Tumor}(T_{a'}^{PB} \leftrightarrow T_{a'}^{Tumor}),$$

where T_Y^X denotes the number of CD8+ T-cells in the subpopulation *Y* (resting, "r"; active, "a", or post-activated, "a' ") in the tissue *X* (PB = peripheral blood; SP = spleen; LN = lymph nodes; BM = bone marrow; or tumor), with population kinetic terms shown for cell generation (Gen), proliferation (Prolif), apoptosis (Apop), activation and deactivation (de/act), and intercompartmental traffic described below. Generation of resting PB T-cells is modeled only to account for reconstitution when number of T-cells falls below homeostatic levels: $\text{Gen} = T_{r,ref}^{PB} * (\max(0, 1 - \frac{T_r^{PB}+T_a^{PB}+T_{a'}^{PB}}{T_{r,ref}^{PB}})^2)$, where $T_{r,ref}^{PB}$ denotes the number of PB resting T-cells at baseline. Similarly, apoptosis of resting PB T-cells is modeled to occur only when numbers exceed homeostatic levels: $\text{Apop} = f_{apop}^{Tr}.\, k_{apop}^{Ta} * (\max(0, T_r^{PB} - T_{r,ref}^{PB}))$, where k_{apop}^{Ta} is the apoptosis rate constant and $f_{apop}^{Tr} < 1$. Activated T-cells undergo activation-induced cell death given by $\text{Apop} = k_{apop}^{Ta} * (\, T_a^{PB} + \frac{f_{AICD}\cdot(T_a^{PB})^2}{T_{r,ref}^{PB}})$. Post-activated T-cells undergo constitutive first-order apoptosis. Proliferation of each activated T-cells occurs at rate Prolif =

$f_{prolif}^{Ta}.k_{prolif}^{T}.T_a^{PB}$, and is assumed to result in 1 activated and 1 post-activated daughter cell, to allow for some serial activity. Thus, each T-cell proliferation event maintains the activated population (no change) and increases the post-activated T-cell number. For traffic, and inter-state conversion, each arrow shows whether the corresponding reaction is a unidirectional or bidirectional reaction, e.g. for $T_{a\prime}^{PB}$, deactivation to T_r^{PB} is unidirectional whereas activation to T_a^{PB} is bidirectional. Resting T-cells are generated in the thymus, mature and traffic to PB, and undergo apoptosis. Additionally, T-cells traffic from PB to other tissues and vice versa and the rate of cell trafficking is determined by tissue-specific partition coefficients, e.g. the following equation shows the trafficking of resting T-cells from PB to spleen, governed by the partition coefficient K_{Tr}^{SP}, defined as the steady state concentration of resting T-cells in spleen divided by the steady state concentration of resting T-cells in PB.

Traffic of resting T-cells from peripheral blood to spleen:

$$\text{Traffic to SP } (T_r^{PB} \leftrightarrow T_r^{SP})$$

$$= k_{exit}^{Tr}.V_{PB}\left(\left(1 + f_{inj}.E_{inj} + f_{drug}.E_{drug}\right).\frac{T_r^{PB}}{V_{PB}}.K_{Tr}^{SP} - \frac{T_r^{SP}}{V_{SP}}\right).$$

In the absence of the drug, CD8+ T-cells in all the tissues are at equilibrium. When the drug or vehicle (placebo) is injected, a very rapid decline in the number of T-cells in the peripheral blood is observed followed by a gradual recovery. This phenomenon of T-cell margination is presumably due to endothelial adhesion/transmigration of PB T-cells that are transiently stressed or partially-activated by injection. This post-treatment margination, adhesion, and efflux effect has been observed with other therapies such as IL2 and IL15 [1-3]. In the model, this is represented semi-mechanistically by transient post-injection augmentation of cell trafficking from PB to

tissue with the terms $f_{inj}.E_{inj}$ and $f_{drug}.E_{drug}$, which respectively represent injection- and drug-induced margination signals that peak immediately upon injection to relative magnitudes f_{inj} and f_{drug} ($f_{inj} \ll f_{drug}$) and decline exponentially thereafter ($E_{inj} = E_{drug} = \exp(-kt)$). All parameters are tuned to match observed placebo and drug-induced T-cell margination (**Error! Reference source not found.**A-C and Supplementary Figure 2A; see Supplementary Table 2 for parameter values). The rate of cell trafficking for T_r^{PB} to other lymphoid tissues or the tumor compartment takes a similar mathematical form, with the use of appropriate tissue-specific partition coefficient. For cell trafficking, we assume the post-activated T-cells traffic similarly to resting T-cells and hence use the same cell trafficking rate constant and partition coefficients, whereas for activated T-cells, we use a higher cell trafficking rate constant ($k_{exit}^{Ta} > k_{exit}^{Tr}$) and also a greater partitioning to tissues, with tissue-specific partition coefficients are multiplied by a factor greater than one ($K_{Ta}^{SP} = f_{Tap}.K_{Tr}^{SP}$; $f_{Tap} > 1$).

In the presence of mosunetuzumab, resting T-cells and post-activated T-cells can become fully activated in a drug-dependent, target-dependent manner [4]; this implementation was based on *in vitro* activation data that showed a typical saturating dose-response, but with a maximum drug-dependent activation of T-cells that increases with the B:T ratio. Thus, T-cell activation was implemented as the product of two Michaelis-Menten terms that respectively capture the dependence on mosunetuzumab concentration (μg/mL) and the dependence of the maximal activation on the ratio of B-cells to the T-cells available for activation, namely, resting and post-activated T-cells. This formulation subsumes the effects of target abundance and binding, bound complex/synapse geometry, and downstream signaling in a potency-based implementation that reflects available in vitro data. The activation takes the same mathematical form in the presence

of blinatumomab, where the EC50 values for blinatumomab concentration and the $B^{PB}:(T_r^{PB} + T_{a\prime}^{PB})$ ratio are different from those used for mosunetuzumab (anti-CD20/CD3 T-cell dependent bispecific (TDB)). Additionally, activated T-cells can directly transition to resting T-cells or move to an interim state of post-activated T-cells before returning to the resting state. The latter path of transition through a "post-activated" state was introduced to the model when it was found to be necessary for calibration to *in vivo* PB T-cell numbers and fraction of activated cells. In the lymphoid tissues and the tumor compartment, we use similar equations to describe the transitions from $T_r^X \leftrightarrow T_a^X$, $T_{a\prime}^X \leftrightarrow T_a^X$, and $T_{a\prime}^X \rightarrow T_r^X$ with the exception that in both TDB_{act}^X and $Blin_{act}^X$ equations, VmT is multiplied by a factor lower than one, to compensate for the fact the cells are more heterogeneously distributed in the tissue than in the PB and the chance of activation for tissue-resident T-cells is lower. In the bone marrow, the first Michaelis-Menten term in the equations for TDB_{act}^{BM} and $Blin_{act}^{BM}$ are a function of CD20+ (B-cell subpopulation) and CD19+ B-cells (pB- and B-cell subpopulations), respectively. This is because in the presence of mosunetuzumab, only CD20+ B-cells contribute to T-cell activation, whereas in the presence of blinatumomab (Blin), CD19+ cells (with or without CD20 expression) contribute to T-cell activation.

T-cell activation and deactivation rates:

$$\text{Act}\ (T_r^{PB} \leftrightarrow T_a^{PB}) = kTact\big((TDB_{act}^{PB} + Blin_{act}^{PB}).T_r^{PB} - fTadeact.T_a^{PB}\big),$$

$$\text{Act}\ (T_{a\prime}^{PB} \leftrightarrow T_a^{PB}) = kTact\big((TDB_{act}^{PB} + Blin_{act}^{PB}).T_{a\prime}^{PB} - fTadeact.T_a^{PB}\big),$$

$$\text{Deact}(T_{a\prime}^{PB} \rightarrow T_r^{PB}) = fTa0deact.T_{a\prime}^{PB},$$

with activation signals given by:

$$TDB_{act}^{PB} = VmT\left(\frac{\left(\frac{B^{PB}}{T_r^{PB}+T_{a'}^{PB}}\right)^S}{\left(\frac{B^{PB}}{T_r^{PB}+T_{a'}^{PB}}\right)^S + (KmBT_{act}^{TDB})^S}\right)\cdot\left(\frac{\left({TDB_c}/{1000}\right)^{n_{act}^{TDB}}}{\left({TDB_c}/{1000}\right)^{n_{act}^{TDB}} + (K_{act}^{TDB})^{n_{act}^{TDB}}}\right),$$

$$Blin_{act}^{PB} = VmT\left(\frac{\left(\frac{B^{PB}}{T_r^{PB}+T_{a'}^{PB}}\right)^S}{\left(\frac{B^{PB}}{T_r^{PB}+T_{a'}^{PB}}\right)^S + \left(KmBT_{act}^{Blin}\right)^S}\right)\cdot\left(\frac{(Blin_c)^{n_{act}^{Blin}}}{(Blin_c)^{n_{act}^{Blin}} + \left(K_{act}^{Blin}\right)^{n_{act}^{Blin}}}\right).$$

in which the parameter VmT represents the maximal activation signal.

Finally, all subpopulations of T-cells undergo apoptosis, modeled by a first-order term with different apoptosis rate constants. For activated T-cells, there is an additional second-order term for activated cell induce death (AICD) which can involve cell-contact interactions such as FAS/FASL binding. Activated T-cells also undergo proliferation and double over time with the assumption that one daughter cell will remain activated and the other one will be post-activated, to allow for limited serial activity, and hence the proliferation term in the equation for post-activated T-cells.

CD19+ and CD20+ B-cell dynamics

Throughout B-cell development, CD19 and CD20 are co-expressed on the surface of all B-cells beginning at the pre-B phase as they mature until B-cell transition to plasma cells. However, a population of pro-B-cells express CD19 but not CD20. In the model, B-cells are generated in the bone marrow, where we track two populations of B-cells: CD19+CD20- pB-cells and

CD19+CD20+ B-cells. The following equations characterize the dynamics of these two subpopulations in the BM and of total B-cells in the PB:

BM and PB B-cell dynamics:

$$\frac{dpB^{BM}}{dt} = \text{Gen} - \text{Apop} + \text{Prolif} - \text{Maturation}\ (pB^{BM} \rightarrow B^{BM}) - \text{Kill}(pB^{BM}),$$

$$\frac{dB^{BM}}{dt} = \text{Maturation}\ (pB^{BM} \rightarrow B^{BM}) - \text{Apop} + \text{Prolif}$$

$$-\text{Traffic to PB}\ (B^{BM} \rightarrow B^{PB}) - \text{Kill}\ (B^{BM}),$$

$$\frac{dB^{PB}}{dt} = \text{Traffic}\ (B^{BM} \rightarrow B^{PB}) + \text{Prolif} - \text{Kill}(B^{PB})$$

$$-\text{Traffic to SP}\ (B^{PB} \rightarrow B^{SP}) - \text{Traffic to LNs}(B^{PB} \rightarrow B^{LN})$$

Where pB^{BM} and B^{BM} denote the number of CD19+CD20- and CD19+CD20+ B-cells in the BM and B^X represents the number of B-cells in in the tissue *X* (as above for T-cells). Cell kinetic terms follow the terminology defined above for T-cells, with the addition of terms for the maturation and cytolytic killing (Kill) of B-cells by T-cells. CD19+CD20- pB-cells are generated in the bone marrow: $\text{Gen} = pB^{BM}_{ref}.\left(k^B_{apop} + k^B_{mat}\right).(1 + \alpha(\max(0,1 - \frac{B^{PB}+B^{SP}+B^{LN}}{B^{PB}_{ref}+B^{SP}_{ref}+B^{LN}_{ref}})))$, where k^B_{apop} and k^B_{mat} are rate constants for B-cell apoptosis and maturation, respectively, and $\frac{B^{PB}+B^{SP}+B^{LN}}{B^{PB}_{ref}+B^{SP}_{ref}+B^{LN}_{ref}}$ provides a normalized B-cell signal to promote B-cell generation in BM when B-cells are depleted in other tissues. They undergo proliferation, $\text{Prolif} = pB^{BM}_{ref}.k^B_{prolif}.(\max(0,1 - \frac{pB^{BM}}{pB^{BM}_{ref}}))$ and apoptosis, $\text{Apop} = k^B_{apop}.pB^{BM}$, and mature to become CD19+CD20+ B2 cells ($\text{Maturation} = k^B_{mat}.pB^{BM}$). CD19+CD20+ B-cells also

undergo proliferation and apoptosis, with similarly formulated terms. In the bone marrow, the ratio of CD19+CD20- pB-cells to CD19+CD20+ B-cells is approximately 1:4. In the model, only the matured B-cells traffic to the PB in a unidirectional manner described by the following equation,

B-cell traffic from BM to PB:

$$\text{Traffic to PB}(B^{BM} \rightarrow B^{PB}) = k_{exit}^{B}.V_{PB}.max\left(0,\frac{B^{BM}}{V_{BM}} - \frac{B^{PB}}{V_{PB}}.K_{B}^{BM}\right).$$

where the rate of cell trafficking is determined by the partition coefficient K_B^{BM}, defined as the steady state concentration of CD19+CD20+ B-cells in BM divided by the steady state concentration of B-cells in PB. Once B-cells are in the PB, they traffic to other lymphoid tissues, such as spleen and lymph nodes, which is modeled by equations similar to the one mentioned above, where the direction of B-cell trafficking is from PB to tissues. Note that in the absence of the drug, B-cells in all the tissues are at equilibrium. For the tumor compartment, unlike the T-cells, we assume that there is no B-cell trafficking to replenish malignant B-cells and the tumor grows only by undergoing proliferation.

In the presence of mosunetuzumab, activated T-cells can kill B-cells in a drug-dependent, target-dependent manner; this was implemented as the product of two Michaelis-Menten equations corresponding to 1) the mosunetuzumab concentration (µg/mL) and 2) the ratio of activated T-cells to B-cells. The killing takes the same mathematical form in the presence of blinatumomab, where the EC50 values for blinatumomab concentration and the $T_a^{PB}:B^{PB}$ ratio are different. In the lymphoid tissues and the tumor compartment, we use similar equations to describe the killing of B-cells by the activated T-cells with two exceptions: 1) VmB is multiplied by a factor lower

than one, and 2) in both TDB_{kill}^{X} and $Blin_{kill}^{X}$ equations, $KmTB_{kill}^{TDB}$ and $KmTB_{kill}^{Blin}$ are multiplied by a factor greater than one; both changes are to compensate for the fact the tissues are not well-stirred environments like PB and the probability of killing of tissue-resident B-cells by activated T-cells is lower. In the bone marrow, the first Michaelis-Menten term in the equations for TDB_{kill}^{BM} and $Blin_{kill}^{BM}$ are a function of CD20+ (B-cell subpopulation) and CD19+ B-cells (pB- and B-cell subpopulations), respectively, and therefore, in the presence of mosunetuzumab, only CD20+ B-cells are killed by activated T-cells, whereas in the presence of blinatumomab, CD19+ B-cells (with or without CD20 expression) are killed.

$$\mathrm{Kill}(B^{PB}) = \ kBkill.(TDB_{kill}^{PB} + Blin_{kill}^{PB}).B^{PB}$$

$$TDB_{kill}^{PB} = VmB\left(\frac{\left(\frac{T_a^{PB}}{B^{PB}}\right)^{n_{kill}^{TDB}}}{\left(\frac{T_a^{PB}}{B^{PB}}\right)^{n_{kill}^{TDB}} + (KmTB_{kill}^{TDB})^{n_{kill}^{TDB}}}\right) \cdot \left(\frac{TDB_c/_{1000}}{TDB_c/_{1000} + K_{kill}^{TDB}}\right)$$

$$Blin_{kill}^{PB} = VmB\left(\frac{\left(\frac{T_a^{PB}}{B^{PB}}\right)^{n_{kill}^{Blin}}}{\left(\frac{T_a^{PB}}{B^{PB}}\right)^{n_{kill}^{Blin}} + \left(KmTB_{kill}^{Blin}\right)^{n_{kill}^{Blin}}}\right) \cdot \left(\frac{Blin_c}{Blin_c + K_{kill}^{Blin}}\right)$$

in which the parameter VmB represents the maximal killing rate.

Pharmacokinetics (PK) of mosunetuzumab and blinatumomab

A standard two-compartment PK model with non-specific clearance (captured by a linear term) was used to describe the measured concentration of mosunetuzumab in serum in cynomolgus monkeys as shown below:

$$\frac{dTDB_{c\,(ug/kg)}}{dt} = -CL_{TDB}\frac{TDB_{c\,(ug/kg)}}{Vc_{TDB}} - CLd_{TDB}\left(\frac{TDB_{c\,(ug/kg)}}{Vc_{TDB}} - \frac{TDB_{p\,(ug/kg)}}{Vp_{TDB}}\right)$$

$$\frac{dTDB_{p\,(ug/kg)}}{dt} = CLd_{TDB}\left(\frac{TDB_{c\,(ug/kg)}}{Vc_{TDB}} - \frac{TDB_{p\,(ug/kg)}}{Vp_{TDB}}\right)$$

$$TDB_c = \frac{TDB_{c\,(ug/kg)}}{Vc_{TDB}}$$

where $TDB_{c\,(ug/kg)}$ and $TDB_{p\,(ug/kg)}$ represent the amount of drug in the central, and peripheral compartments with the units being μg/kg. Parameters Vc_{TDB} and Vc_{TDB} represent the volume of central and peripheral compartments, CL_{TDB} represents the clearance from the central compartment, and CLd_{TDB} denotes the distribution clearance between the central and peripheral compartments. The units for volume and clearance terms are mL/kg and mL/day/kg, respectively. TDB_c denotes the drug concentration in the central compartment; the concentration of mosunetuzumab in other tissues is assumed to be TDB_c multipled by a tissue-specific partition coefficient with a value lower than one [5]. Note that we used the actual PK measurements as the forcing function when we performed model calibration and generated virtual cohort of cynos. The PK model with allometric scaling (exponent for CL = 0.85) was only used to project the human PK profiles and predict the results in patients.

For the PK of blinatumomab, we used the model in [6], where blinatumomab is administered as continuous infusion to patients and is rapidly cleared from circulation:

$$\frac{dBlin_{c\,(ug)}}{dt} = \frac{-CL_{Blin}.\,Blin_{c\,(ug)}}{Vz_{Blin}},$$

$$Blin_c = \frac{Blin_{c\,(ug)}}{Vz_{Blin}}.\,1000$$

$Blin_{c\,(ug)}$ and $Blin_c$ denote the systemic amount (ug) and concentration (ng/mL) of the drug, respectively, and we used the same tissue-specific partition coefficients that we used for mosunetuzumab to estimate the tissue concentration of blinatumomab.

Cytokine dynamics

In the model, cytokine production is assumed to be the consequence of T-cell activation. In the presence of blinatumomab or mosunetuzumab, cytokines are produced by (or induced by) locally activated T-cells that receive an instantaneous local cytokine production signal, which is determined by drug concentration and decay in the number of target cells. For example, the PB IL6 levels is governed by following equation.

$$\frac{dIL6^{PB}}{dt} = k_{prod}^{IL6}\frac{T_a^{PB}}{V_{PB}}\left(\left(\frac{(TDB_c)^{n_{act}^{TDB}}}{(TDB_c)^{n_{act}^{TDB}} + (K_{act}^{TDB})^{n_{act}^{TDB}}} + \frac{(Blin_c)^{n_{act}^{Blin}}}{(Blin_c)^{n_{act}^{Blin}} + \left(K_{act}^{Blin}\right)^{n_{act}^{Blin}}}\right)\frac{B^{PB}}{B_{ref}^{PB}}\right) - k_{deg}^{IL6}.\,IL6^{PB},$$

where k_{prod}^{IL6} and k_{deg}^{IL6} represent production and degradation rate of IL6 and the activated T-cells that receive the local production signal (the term in the big parentheses) produce IL6. The variable B^{PB} denotes the instantaneous number of B-cells in PB, while B_{ref}^{PB} denotes the number

of PB B-cells at baseline. Tissue and tumor IL6 levels are governed by similar mathematical equations. Finally, the measured IL6 in serum is the sum of IL6 produced in the peripheral blood and the contribution from tissue IL6, as shown below:

$$IL6^{tot} = IL6^{PB} + f_{tiss}^{IL6}\left(\frac{IL6^{SP}.V_{SP} + IL6^{LN}.V_{LN} + IL6^{BM}.V_{BM} + IL6^{tumor}.V_{tumor}}{V_{PB}}\right),$$

where f_{tiss}^{IL6} represents contribution of tissue IL6 to the measured IL6 levels.

Supplementary Method 2. Technical Workflow of the QSP Model Development

Calibration of a Reference Virtual Cynomolgus Monkey

We used a scatter search methodology to calibrate adjustable model parameters to match the preclinical measurements in cynos. We defined the objective function as the sum of the difference between simulation results and the preclinical data for circulating CD8+ T-cell numbers, B-cell numbers, and percentage of CD8+CD69+ T-cells and limited B:T ratios in spleen and lymph nodes (only one data point after the last dose) across all animals in the three dosing groups as well as the placebo group. The outcome of the calibration is a single parameter set that best described the observed data, and the model structure together with this parameter set is termed the "reference virtual cyno". The search space for parameter values was restricted to biologically relevant ranges and consistent with the *in vitro* data and previously published measurements (see Supplementary Table 2 for list of parameters, values and references). In our optimization, we used the pre-treatment levels of B-cells and CD8+ T-cells for each individual animal to set the initial conditions. Due to large variability in the data and additional impact of ADA on PK profiles, we used the actual PK data (Supplementary Figure 1) for each animal as a forcing function to calibrate the model. For ADA-positive data points, we used the measured concentration if it was higher than the level of quantification (LOQ) for the PK assay; for data points below LOQ, the concentration was estimated using the estimated slope of the PK profile.

Generation of a Virtual Human ALL Patients

The model was adjusted to represent patients with acute lymphoblastic leukemia. We first replaced cyno physiological parameters with the appropriate human physiological volumes and T and B-cell numbers for peripheral blood and lymphoid tissues, preserving the model structure

and other parameter estimates (See Supplementary Table 2 for human numbers). We then added representations of the pharmacokinetics of blinatumomab and its mechanistic effects including activation of CD8+ T lymphocytes, and subsequent killing of target CD19+ B lymphocytes. For blinatumomab PD, published data provided an estimate of concentration-dependent potency and suggested a similar quantitative cell-ratio dependence as was seen for mosunetuzumab [7-10].

Generation of a Virtual Cohort of Cynomolgus Monkeys

To capture the biological variability observed in preclinical data, we generated a cohort of virtual cynos using the method introduced in [11], with three main steps: (1) subject generation: to explore uncertainty by generating parameter sets locally randomized around the initial fit (reference virtual cyno); (2) subject selection: to select those virtual cynos whose simulated profiles lie within the range of preclinical data for circulating and tissue measurements at all dose levels. Additionally, we selected the virtual cynos independent of the initial values for circulating B and T-cell levels and used the average PK concentration for a given dose level, so that for predictions, virtual cynos can be used for different dose levels and regimens (e.g. PK profiles) and a wide range of initial conditions for circulating B and T-cells; and (3) subject validation: to validate the virtual cohort of cynos against another preclinical dataset, not previously used for training.

Generation of a Virtual Population of NHL Patients

To create a virtual population of NHL patients, we translated the virtual population of healthy cynos (with the corresponding biological variability) to a virtual population of healthy humans by using the appropriate human physiological (blood/tissue) compartment volumes and T and B-

cell numbers for peripheral and lymphoid tissues. Then, we added a tumor compartment by implementing a large B-cell dense mass and included additional disease-related variability such as baseline peripheral B and T-cells [12], tumor load, tumor cell doubling time (2-6 weeks) and a higher range of B:T ratio in the tumor microenvironment as B-cells are assumed to be the majority of cells in the tumor microenvironment. We then selected the NHL virtual population by selecting virtual patients such that simulated treatment with blinatumomab matched clinical data; i.e., we used the PK of blinatumomab and its downstream effects on T-cell activation and B-cell killing to simulate effects of blinatumomab and selected virtual patients such that the distribution of anti-tumor efficacy matched the efficacy data observed in clinical study in which r/r DLBCL patients received continuous infusion of blinatumomab for 8 weeks using a step-up dosing regimen (5-15-60 μg/m^2/day for 8 weeks) and their tumor size was evaluated at week 10.

Supplementary Figures

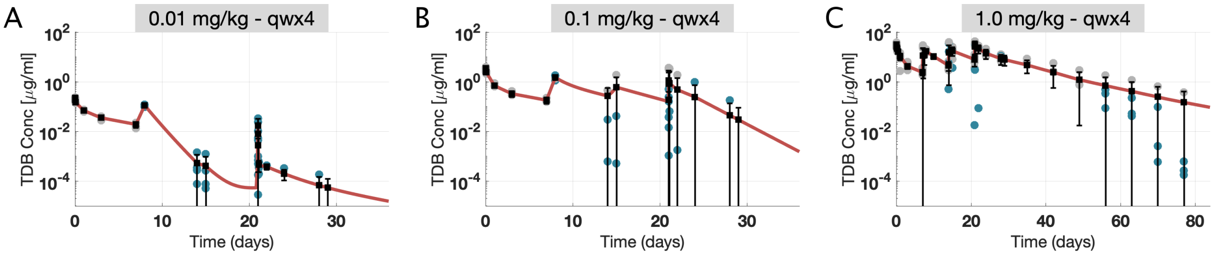

Supplementary Figure 1. Time Profiles of Mosunetuzumab Serum Concentration in Different Dosing Groups in the Multiple Dose IV Injection Study (Preclinical Study 1)

The PK data suggests that mosunetuzumab exposure was confirmed and maintained in the high dose group (1 mg/kg). In the low (0.01 mg/kg) and medium (0.1 mg/kg) dose groups, mosunetuzumab exposure was confirmed, however not maintained over the course of the study, due to the presence of ADA. After the first dose, serum concentrations decreased similarly for the first 7 days post-dose for low, medium and high dose groups. One day after the second dose, mosunetuzumab serum concentrations increased for all dose groups. However, for all animals in the low dose group and 3 out of 4 animals in the medium dose group, serum concentrations at subsequent time points after the second dose decreased significantly. This overall decline and trend of lower than expected serum concentrations is indicative of the presence of ADA having an effect on the PK of mosunetuzumab in preclinical studies. Subsequent analysis showed that these animals with the decreased serum concentrations were in fact ADA positive. Gray and blue dots show individual ADA- and ADA+ data points, respectively (n = 4 for 0.01 and 0.1 mg/kg; total n = 11 for 1 mg/kg with n = 4 after D29 in the recovery phase), and vertical black lines show mean +/- standard deviation. In our optimization, we used the measured PK for each animal as a forcing function to calibrate the model. This was done by a linear interpolation of log-transformed PK data points measured for each animal. For ADA-positive data points, we used the measured concentration if it was higher than the level of quantification (LOQ) for the

PK assay; for data points below LOQ, the concentration was estimated using the estimated slope of the PK profile. Red curves are the average of reconstructed PK profiles for each dose group.

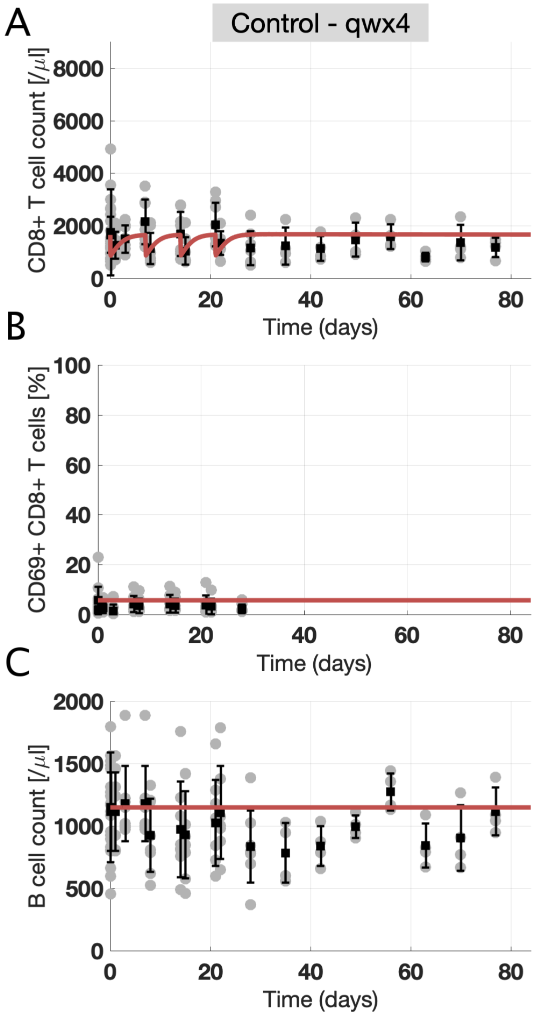

Supplementary Figure 2. Time Profile Data and Model Fits for Measurements in PB in the Multiple Dose Study of Control Vehicle in Cynomolgus Monkeys (Preclinical Study 1)

The reference virtual cyno model replicates T and B-cell dynamics in the peripheral blood for the cynos dosed with the vehicle control, given weekly for four weeks. Each row shows one measurement in circulation (top: CD8+ T-cells; middle: percentage of activated T-cells (CD8+CD69+ T-cells); and bottom: B-cells). Gray dots show individual data points (total n = 8

in the control group with n = 4 after D29 in the recovery phase) and vertical black lines show mean +/- standard deviation. Red curves are the calibrated model outputs.

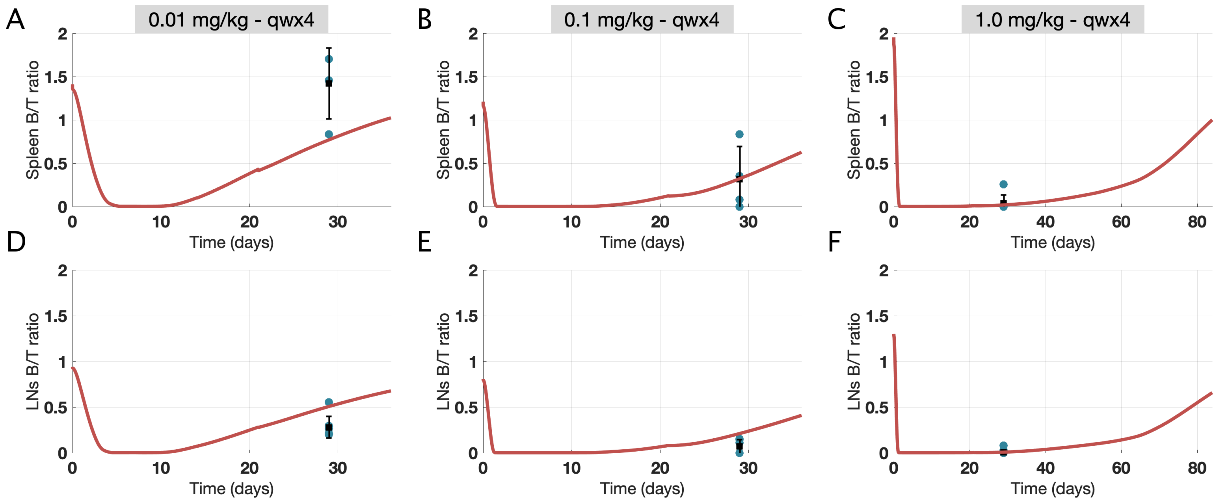

Supplementary Figure 3. Time Profile Data and Model Fits for Measurements in Lymphoid Tissues in the Multiple Dose Study of Mosunetuzumab in Cynomolgus Monkeys (Preclinical Study 1)

The reference virtual cyno model replicates dynamics of B:T ratios in spleen and LNs for different dose levels of mosunetuzumab in cynos. Each column shows a different dose level, ranging from 0.01 mg/kg to 1 mg/kg given weekly for four weeks. Each row shows T:B ratios in one lymphoid tissue (top: spleen; and bottom: LNs). Gray and blue dots show individual ADA- and ADA+ data points, respectively (n = 4 for 0.01 and 0.1 mg/kg; total n = 7 for 1 mg/kg; for LNs, there are two measurements per animal), and vertical black lines show mean +/- standard deviation. Red curves are the calibrated model outputs.

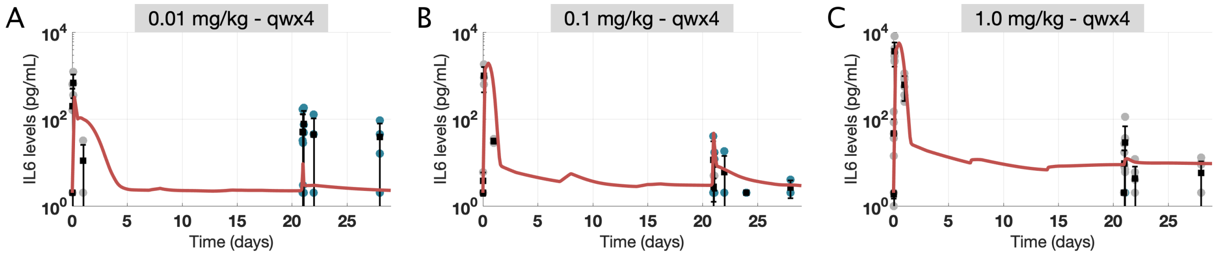

Supplementary Figure 4. Time Profile Data and Model Simulations for Serum IL6 levels in the Multiple Dose Study of Mosunetuzumab in Cynomolgus Monkeys (Preclinical Study 1)

The reference virtual cyno model replicates dynamics of cytokine levels for different dose levels of mosunetuzumab in cynos. Each column shows a different dose level, ranging from 0.01 mg/kg to 1 mg/kg given weekly for four weeks. Gray and blue dots show individual ADA- and ADA+ data points, respectively (n = 4 for 0.01 and 0.1 mg/kg; total n = 7 for 1 mg/kg), and vertical black lines show mean +/- standard deviation. Red curves are the model outputs.

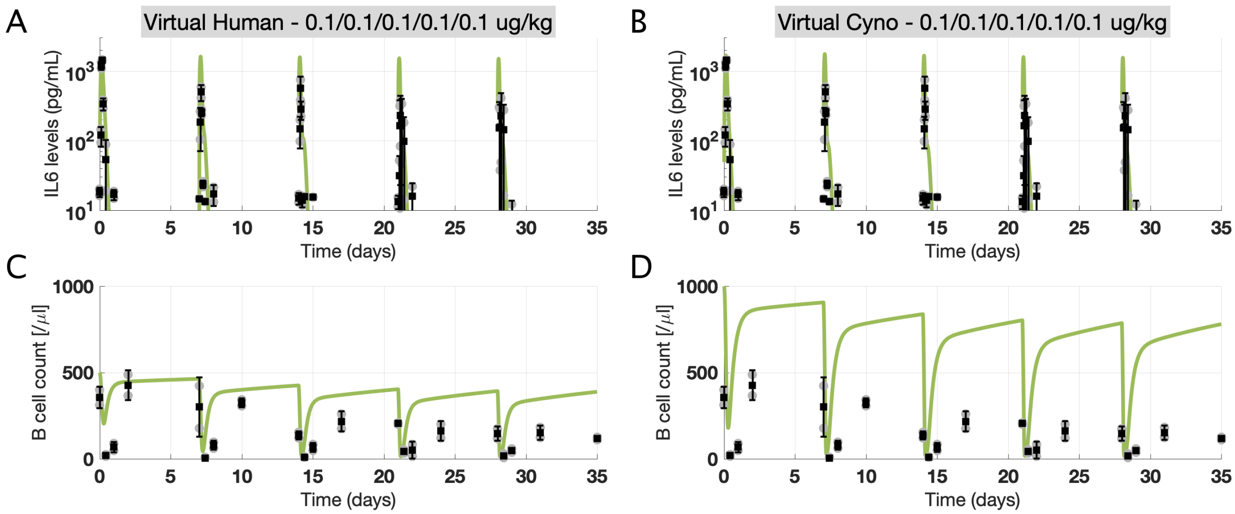

Supplementary Figure 5. Time Profiles of IL6 and B-Cell Levels in Chimpanzees Treated with Blinatumomab (Preclinical Study 4)

The reference virtual human (left column) and virtual cyno (right column) qualitatively predict dynamics of IL6 levels and B-cells for a multi-dose study of blinatumomab in chimpanzees (0.1 ug/kg given weekly for five weeks). Gray dots show individual data points (n = 2) and vertical black lines show mean +/- standard deviation. Green curves are predictions of the reference virtual human and cyno.

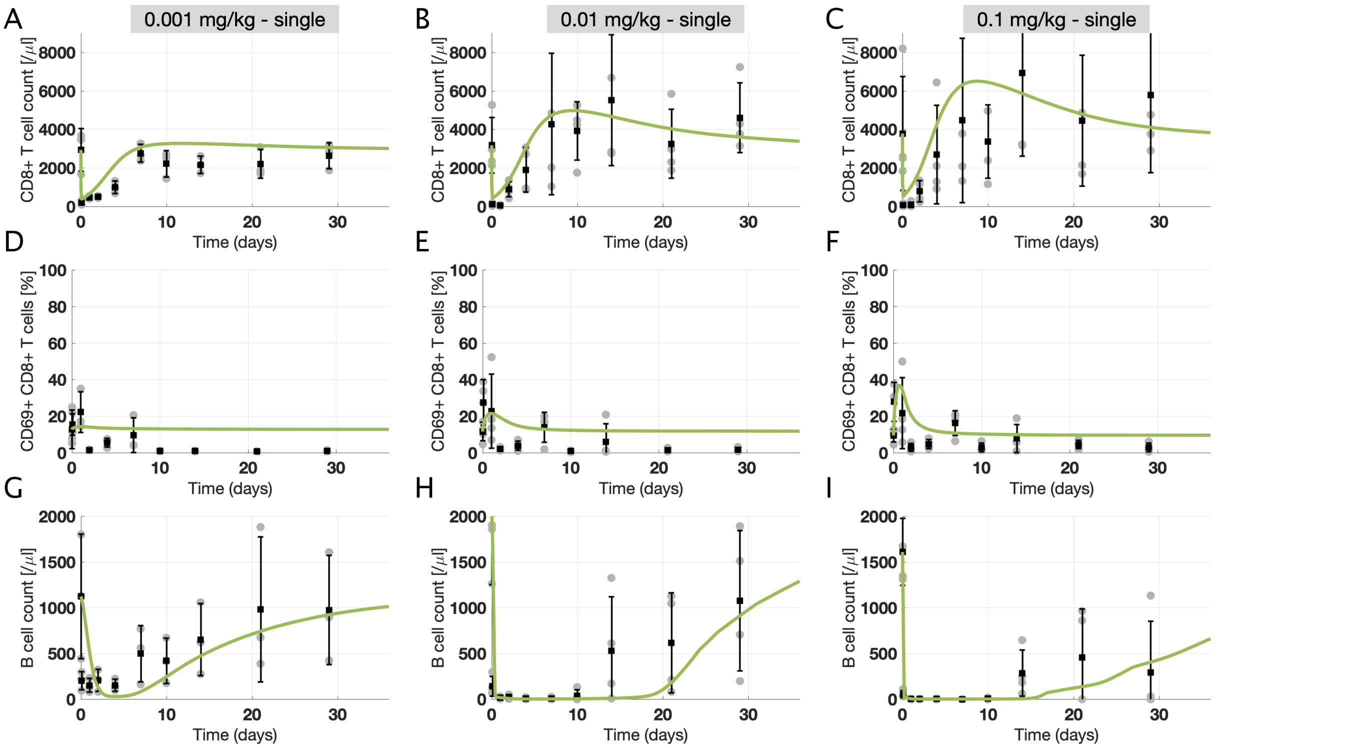

Supplementary Figure 6. Reference Virtual Cyno Predictions for Measurements in PB in the Single Dose Study of Mosunetuzumab in Cynomolgus Monkeys (Preclinical Study 2)

The predictions of reference virtual cyno model closely match T and B-cell dynamics in the peripheral blood for different dose levels of mosunetuzumab in cynos. Initial B and T-cells from each animal and the actual PK was used to run the model. Each column shows a different dose level, ranging from 0.001 mg/kg to 0.1 mg/kg given as a single injection. Each row shows a single measurement in circulation (top: CD8+ T-cells; middle: percentage of activated T-cells (CD8+CD69+ T-cells); bottom: B-cells). Gray dots show individual data points (n = 3 for 0.001 mg/kg; n = 4 for 0.01 and 0.1 mg/kg) and vertical black lines show mean +/- standard deviation. Green curves are predictions of the reference virtual cyno.

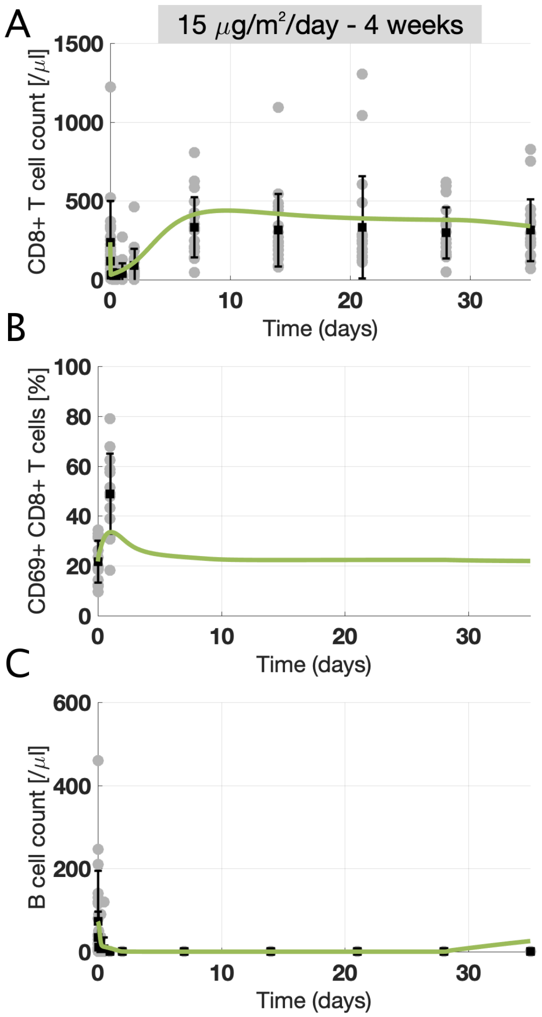

Supplementary Figure 7. Translated Human ALL Model Predictions for Measurements in PB in the Clinical Study of Blinatumomab Given via Continuous Infusion to Patients with ALL (Clinical Study 1)

The reference virtual cyno model was translated to human ALL model by making appropriate changes in physiological volumes, B and T-cell numbers of PB and lymphoid tissues and by including blinatumomab PK and its downstream effects. The virtual ALL patient successfully predicts T and B-cell dynamics in the peripheral blood for continuous infusion of blinatumomab given to ALL patients at 15 µg/m2/day for four weeks. Initial B and T-cells in PB from each patient was used to run the model. Each row shows a single measurement in circulation (top:

CD8+ T-cells; middle: percentage of activated T-cells (CD8+CD69+ T-cells); and bottom: B-cells). Gray dots show individual data points (n = 20) and vertical black lines show mean +/- standard deviation. Green curves are predictions of the virtual ALL patient.

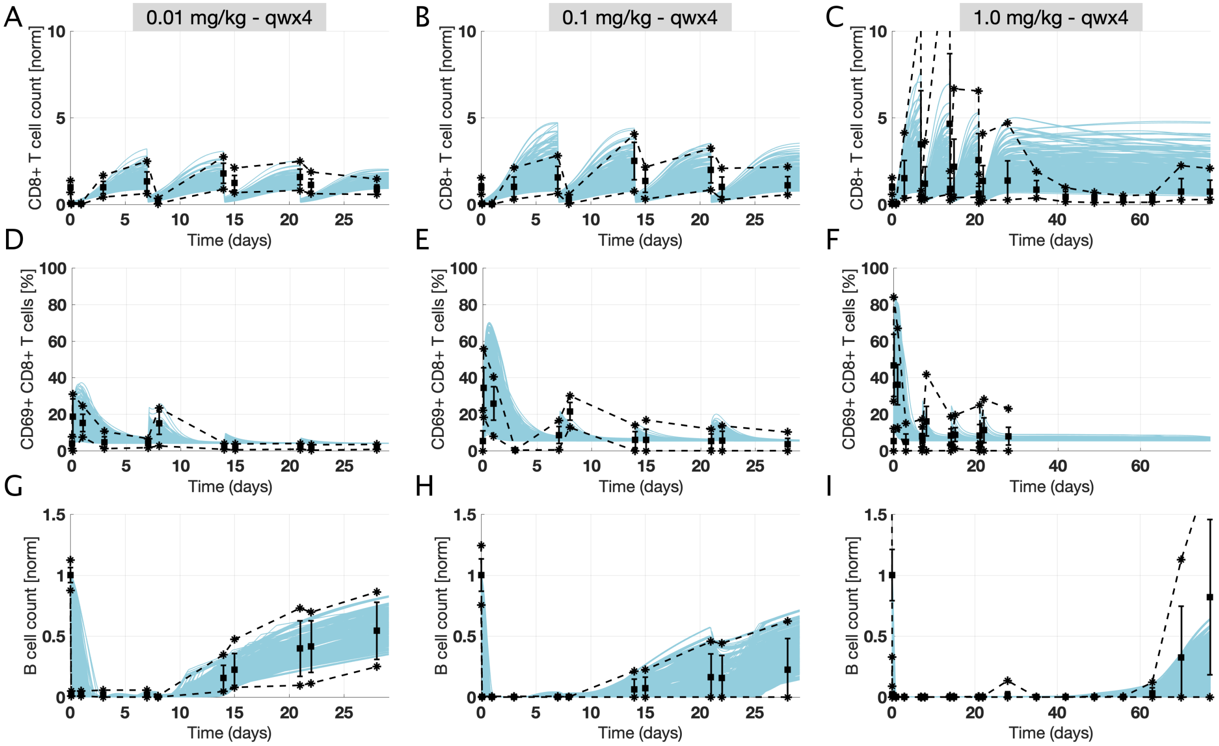

Supplementary Figure 8. Time Profiles of Virtual Cohort of Cynos Capture the Observed Variability in PB in the Multiple Dose Study of Mosunetuzumab in Cynomolgus Monkeys (Preclinical Study 1)

The virtual cohort of cynos are shown to capture the range of observed variability in T and B-cell profiles in the peripheral blood for different dose levels of mosunetuzumab in cynos. Each column shows a different dose level, ranging from 0.01 mg/kg to 1 mg/kg given weekly for four weeks. Each row shows a single measurement in PB (top: normalized CD8+ T-cells; middle: percentage of activated T-cells (CD8+CD69+ T-cells); and bottom: normalized B-cells). Black asterisks indicate the range of observed data and vertical black lines show mean +/- standard deviation. Blue curves are the virtual cynos in the cohort.

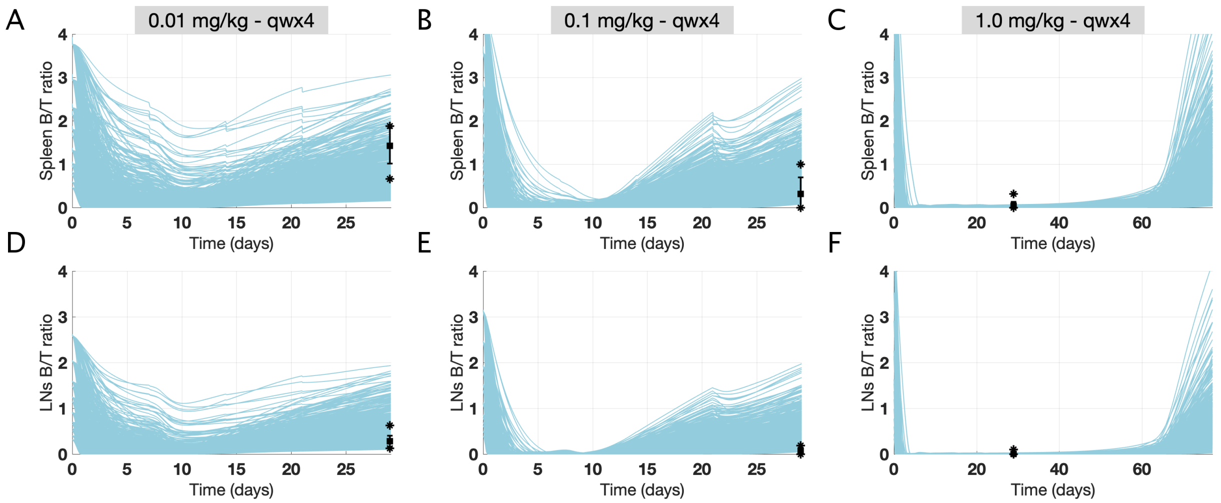

Supplementary Figure 9. Time Profiles of Virtual Cohort of Cynos Capture the Observed Variability in Lymphoid Tissues in the Multiple Dose Study of Mosunetuzumab in Cynomolgus Monkeys (Preclinical Study 1)

The virtual cohort of cynos are shown to capture the range of observed variability in B:T ratios in spleen and LNs for different dose levels of mosunetuzumab in cynos. Each column shows a different dose level, ranging from 0.01 mg/kg to 1 mg/kg given weekly for four weeks. Each row shows T:B ratios in one lymphoid tissue (top: spleen; and bottom: LNs). Black asterisks indicate the range of observed data and vertical black lines show mean +/- standard deviation. Blue curves are the virtual cynos in the cohort.

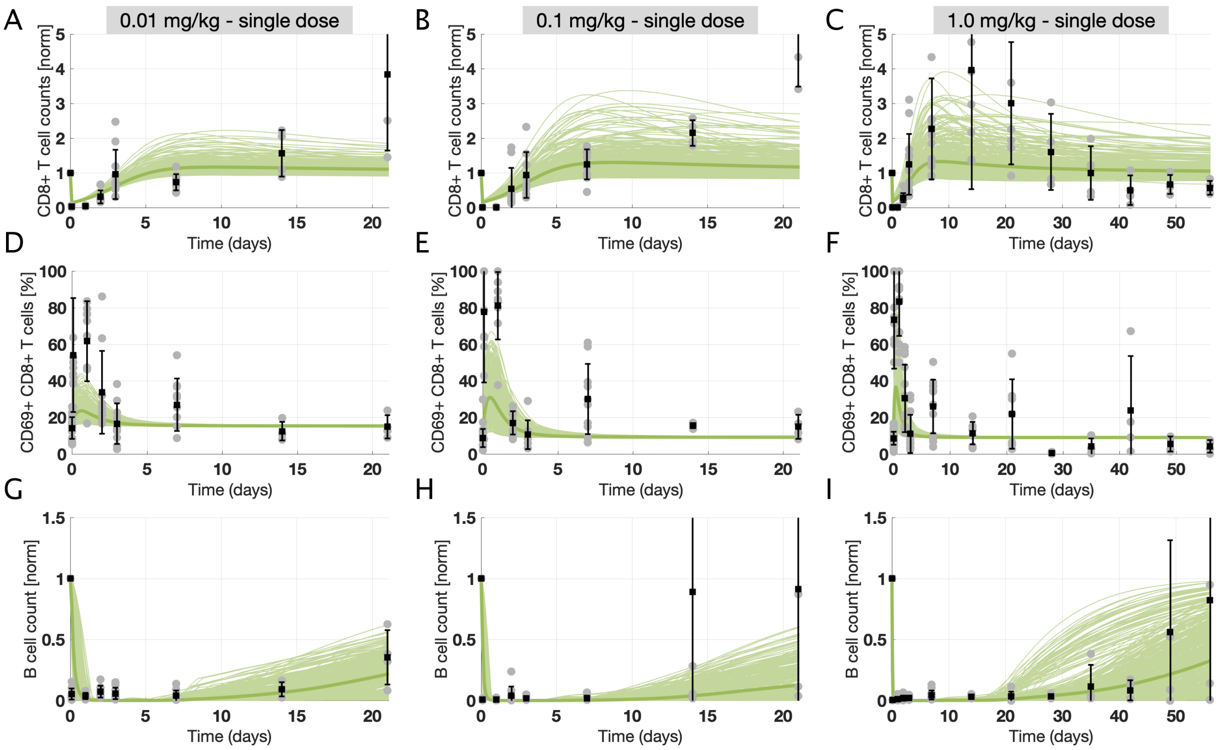

Supplementary Figure 10. Virtual Cohort of Cynos Predicts the Observed Variability in PB in the Single Dose Study of Mosunetuzumab in Cynomolgus Monkeys (Preclinical Study 3)

The virtual cohort of cynos successfully predicts T and B-cell dynamics in the peripheral blood for different dose levels of mosunetuzumab in cynos. Each column shows a different dose level, ranging from 0.01 mg/kg to 1 mg/kg given as a single injection. Each row shows a single measurement in circulation (top: normalized CD8+ T-cells; middle: percentage of activated T-cells (CD8+CD69+ T-cells); and bottom: normalized B-cells). Gray dots show individual data points (total n = 10 for 0.01 and 0.1 mg/kg with n = 4 after D7; total n = 13 for 1 mg/kg with n = 7 after D7 and n = 3 after D21 in the recovery phase) and vertical black lines show mean +/- standard deviation. Green curves are predictions of the virtual cynos in the cohort.

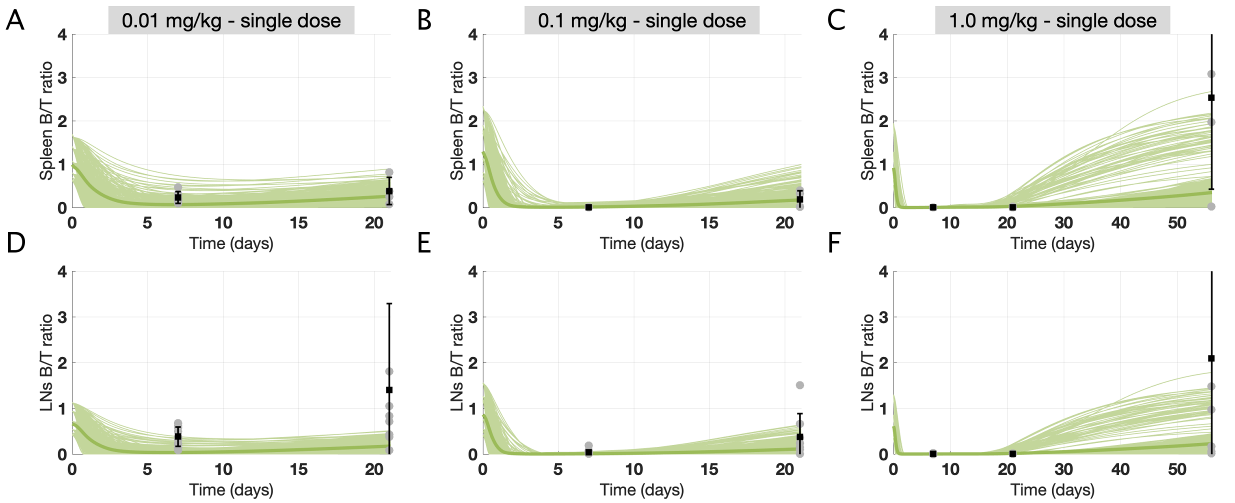

Supplementary Figure 11. Virtual Cohort of Cynos Predicts the Variability in Lymphoid Tissues in the Single Dose Study of Mosunetuzumab in Cynomolgus Monkeys (Preclinical Study 3)

The virtual cohort of cynos successfully predicts the range of observed variability in B:T ratios in spleen and LNs for different dose levels of mosunetuzumab in cynos. Each column shows a different dose level, ranging from 0.01 mg/kg to 1 mg/kg given a single injection. Each row shows T:B ratios in one lymphoid tissue (top: spleen; and bottom: LNs). Gray dots show individual data points (n = 6 for all doses at D7; n = 4 for all dose at D21; n = 3 for 1.0 mg/kg at D56; for LNs, there are two measurements per animal) and vertical black lines show mean +/- standard deviation. Green curves are predictions of the virtual cynos in the cohort.

Supplementary Table 1. A Summary of Preclinical and Clinical Studies Used in the Workflow of QSP Model Development

Study Title	Dose levels/regimens	Measurements	Data Source	Used in workflow
Preclinical Study 1: multiple dose study of anti-CD20/CD3 TDB (E. coli produced) via IV injection in cynomolgus monkeys	0.01, 0.1, and 1.0 mg/kg given weekly via slow bolus IV injections for a total of four doses.	PK; multiple PD markers such as B-cells, T-cells, and T-cell activation markers in peripheral blood and lymphoid tissues; PK and PD sampling schedule: 0, 0.0035, 0.0208, 0.083, 0.17, 0.25, 0.5, 1, 3, 6.99, 7.0833, 7.25, 7.5, 8, 10, 13.99, 14.0833, 15, 20.99, 21.0035, 21.0208, 21.083, 22, 24, 28, 29, 35, 42, 49, 56, 63, 70, 77 days	Genentech study	Model calibration; virtual cyno cohort generation
Preclinical Study 2: single dose study of mosunetuzumab via IV injection in cynomolgus monkeys	0.001, 0.01, and 0.1 mg/kg given as a single IV injection	PK; multiple PD markers such as B-cells, T-cells, and T-cell activation markers in peripheral blood; PK and PD sampling schedule: 0, 0.0035, 0.083, 0.25, 1, 2, 4, 7, 10, 14, 21, 29 days	Genentech study	Validation of reference virtual cyno
Preclinical Study 3: single dose study of mosunetuzumab via IV	0.01, 0.1, and 1.0 mg/kg given as a single IV injection	PK; multiple PD markers such as B-cells, T-cells, and T-cell activation markers in	Genentech study	Virtual cyno cohort validation

injection in cynomolgus monkeys		peripheral blood and lymphoid tissues; PK and PD sampling schedule: 0, 0.0208, 0.0833, 0.25, 1, 2, 3, 7, 14, 21, 28, 35, 42, 49, 56 days		
Preclinical Study 4: multiple dose study of anti-CD19/CD3 in chimpanzees via IV infusion	0.1 µg/kg infused weekly over a 2-hour period for a total of five infusions	PK; multiple PD markers such as peripheral cytokines and lymphocytes; see [13] for the sampling schedule;	Published data [13]	Cytokine hypothesis validation
Clinical Study 1: Continuous infusion of anti-CD19/CD3 bispecific antibody in ALL patients	Cont. infusion at a dose level of 15 µg/m^2/day for 4 weeks to 20 evaluable MRD+ ALL patients; subsequent treatment cycles spaced by 2-week treatment-free intervals.	PK; multiple PD markers such as peripheral lymphocytes (B and T-cells); see [6] for the sampling schedule;	Published data [6]	Cyno to human translation and validation
Clinical Study 2: Phase I clinical trial of anti-CD19/CD3-bispecific antibody in NHL patients	Cont. infusion for 4 or 8 weeks at different dosing regimens to 76 patients. Two cohorts of interest: 1) cont. infusion at 60 µg/m^2/day starting at D0; 2) a step-up dosing schedule in which patients received 5, 15,	Assessing the safety and efficacy of blinatumomab including anti-tumor activity, cytokines and PK endpoints; see [12] for the sampling schedule;	Published data [12]	NHL virtual population generation and validation

	60 μg/m²/day at D0, D7, and D14, respectively.			
Clinical Study 3: Phase II clinical trial of anti-CD19/CD3-bispecific antibody in relapsed/refractory DLBCL patients	Cont. infusion using a step-up dosing regimen to 23 patients (9-28-112 μg/day with weekly dose increases). The target dose in all patients was 112 μg/day administered up to 8 weeks, followed by 4 treatment-free weeks.	Assessing efficacy of blinatumomab including anti-tumor activity; among 21 evaluable patients, the overall response rate after one blinatumomab cycle was 43%; see [14] for the sampling schedule;	Published data [14]	NHL virtual population generation and validation

Supplementary Table 2. List of parameters, descriptions and values used for cyno, human, ALL, and DLBCL scenarios

See param_table.xlsx for a complete list of parameters used for cyno, human, ALL, and DLBCL scenarios

Supplementary Data File 1. A zip file containing Matlab SimBiology and .m files to run the model

References

1 Conlon, K. C. *et al.* Redistribution, hyperproliferation, activation of natural killer cells and CD8 T cells, and cytokine production during first-in-human clinical trial of recombinant human interleukin-15 in patients with cancer. *Journal of clinical oncology* **33**, 74 (2015).

2 Atkins, M. B. *et al.* Phase I evaluation of recombinant interleukin-2 in patients with advanced malignant disease. *Journal of Clinical Oncology* **4**, 1380-1391 (1986).

3 Langer, H. F. & Chavakis, T. Leukocyte–endothelial interactions in inflammation. *Journal of cellular and molecular medicine* **13**, 1211-1220 (2009).

4 Sun, L. L. *et al.* Anti-CD20/CD3 T cell–dependent bispecific antibody for the treatment of B cell malignancies. *Science translational medicine* **7**, 287ra270-287ra270 (2015).

5 Shah, D. K. & Betts, A. M. in *MAbs.* 297-305 (Taylor & Francis).

6 Klinger, M. *et al.* Immunopharmacological response of patients with B-lineage acute lymphoblastic leukemia to continuous infusion of T cell-engaging CD19/CD3-bispecific BiTE antibody blinatumomab. *Blood*, blood-2012-2001-400515 (2012).

7 d'Argouges, S. *et al.* Combination of rituximab with blinatumomab (MT103/MEDI-538), a T cell-engaging CD19-/CD3-bispecific antibody, for highly efficient lysis of human B lymphoma cells. *Leukemia research* **33**, 465-473 (2009).

8 Brischwein, K. *et al.* MT110: a novel bispecific single-chain antibody construct with high efficacy in eradicating established tumors. *Molecular immunology* **43**, 1129-1143 (2006).

9 Brischwein, K. *et al.* (American Society of Hematology, 2006).

10 Hoffmann, P. *et al.* Serial killing of tumor cells by cytotoxic T cells redirected with a CD19-/CD3-bispecific single-chain antibody construct. *International journal of cancer* **115**, 98-104 (2005).

11 Gadkar, K. *et al.* A mechanistic systems pharmacology model for prediction of LDL cholesterol lowering by PCSK9 antagonism in human dyslipidemic populations. *CPT: pharmacometrics & systems pharmacology* **3**, 1-9 (2014).

12 Hijazi, Y. *et al.* Pharmacokinetic and Pharmacodynamic Relationship of Blinatumomab in Patients with Non-Hodgkin Lymphoma. *Current clinical pharmacology* **13**, 55-64 (2018).

13 Schlereth, B. *et al.* T-cell activation and B-cell depletion in chimpanzees treated with a bispecific anti-CD19/anti-CD3 single-chain antibody construct. *Cancer Immunology, Immunotherapy* **55**, 503-514 (2006).

14 Viardot, A. *et al.* Phase 2 study of bispecific T-cell engager (BiTE®) antibody blinatumomab in relapsed/refractory diffuse large B cell lymphoma. *Blood*, blood-2015-2006-651380 (2016).
